# CXCL12-mediated HOXB5 overexpression facilitates Colorectal Cancer metastasis through transactivating CXCR4 and ITGB3

**DOI:** 10.7150/thno.52199

**Published:** 2021-01-01

**Authors:** Weibo Feng, Wenjie Huang, Jie Chen, Chenyang Qiao, Danfei Liu, Xiaoyu Ji, Meng Xie, Tongyue Zhang, Yijun Wang, Mengyu Sun, Dean Tian, Daiming Fan, Yongzhan Nie, Kaichun Wu, Limin Xia

**Affiliations:** 1 State key Laboratory of Cancer Biology, National Clinical Research Center for Digestive Diseases and Xijing Hospital of Digestive Diseases, Fourth Military Medical University, Xi'an 710032, Shaanxi Province, China.; 2 Department of Gastroenterology, Institute of Liver and Gastrointestinal Diseases, Hubei Key Laboratory of Hepato-Pancreato-Biliary Diseases, Tongji Hospital of Tongji Medical College, Huazhong University of Science and Technology, Wuhan 430030, Hubei Province, China.; 3 Hepatic Surgery Center, Tongji Hospital, Tongji Medical College, Huazhong University of Science and Technology; Clinical Medicine Research Center for Hepatic Surgery of Hubei Province; Key Laboratory of Organ Transplantation, Ministry of Education and Ministry of Public Health, Wuhan, Hubei, 430030, China.

**Keywords:** homeobox B5, colorectal cancer, metastasis, C-X-C motif chemokine receptor 4, AMD3100

## Abstract

**Background:** Metastasis is the major reason for the high mortality of colorectal cancer (CRC). However, the molecular mechanism underlying CRC metastasis remains unclear. Here, we report a novel role of homeobox B5 (HOXB5), a member of the HOX family, in promoting CRC metastasis.

**Method:** The expression of HOXB5 and its target genes were examined by immunohistochemistry in human CRC. Chromatin immunoprecipitation and luciferase reporter assays were performed to measure the transcriptional regulation of target genes by HOXB5. The metastatic capacities of CRC cells were evaluated by *in vivo* lung and liver metastatic models.

**Results:** The elevated expression of HOXB5 was positively correlated with distant metastasis, higher AJCC stage, and poor prognosis in CRC patients. HOXB5 expression was an independent and significant risk factor for the recurrence and survival in CRC patients. Overexpression of HOXB5 promoted CRC metastasis by transactivating metastatic related genes, C-X-C motif chemokine receptor 4 (CXCR4) and integrin subunit beta 3 (ITGB3). C-X-C motif chemokine ligand 12 (CXCL12), which is the ligand of CXCR4, upregulated HOXB5 expression through the extracellular regulated protein kinase (ERK)/ETS proto-oncogene 1, transcription factor (ETS1) pathway. The knockdown of HOXB5 decreased CXCL12-enhanced CRC metastasis. Furthermore, AMD3100, a specific CXCR4 inhibitor, significantly suppressed HOXB5-mediated CRC metastasis. HOXB5 expression was positively correlated with CXCR4 and ITGB3 expression in human CRC tissues, and patients with positive co-expression of HOXB5/CXCR4, or HOXB5/ITGB3 exhibited the worst prognosis.

**Conclusion:** Our study implicates HOXB5 as a prognostic biomarker in CRC, and defines a CXCL12-HOXB5-CXCR4 positive feedback loop that plays an important role in promoting CRC metastasis.

## Introduction

Colorectal cancer (CRC) is the third most common cancer and the second leading cause of cancer mortality worldwide, which represents a global health burden 1. Although targeted therapy and immunotherapy have shifted the treatment paradigm for CRC in recent years, local or systemic recurrence of CRC still remain unresolved obstacles in clinical practice 2. Hence, further defining the molecular mechanisms of CRC metastasis presents a great opportunity to develop novel therapies against metastatic CRC.

The Homeobox (HOX) family of transcription factors are key regulators of embryonic development, tissue homeostasis and many other aspects of cellular physiology 3. In the past decades, extensive evidence has accumulated to indicate that dysregulation of HOX genes facilitates cancer progression through various mechanisms 3. Human HOX family comprises 39 proteins belonging to 4 separate clusters (HOXA, HOXB, HOXC and HOXD) 4. The HOXB cluster consists of 10 genes and the alteration of their expression has been shown in cancer 4. Intriguingly, most HOXB genes function as oncogenes and are upregulated in a broad spectrum of human cancers. For example, HOXB5 is implicated in multiple human cancers including breast cancer 5, pancreatic cancer 6, retinoblastoma 7, gastric cancer 8, non-small cell lung cancer 9 and head and neck squamous cell carcinoma 10. However, little is known about its role in CRC metastasis.

For most solid tumors, the metastatic cascade begins with cancer cells breaching out their microenvironment where they interact with immune cells. Novel therapeutic approaches targeting the interaction between cancer cells and their microenvironment have shown promising results in many cancer types. Recently, the chemokine CXCL12 and its receptor CXCR4 have emerged as promising drug targets due to their crucial roles in the tumor microenvironment and trafficking of immune cells 11. CXCR4 is an evolutionarily conserved transmembrane G-protein coupled receptor (GPCR) that selectively binds to its soluble ligand CXCL12 (SDF-1) 12. CXCR4 signaling facilitates tumor metastasis in tissues where CXCL12 is abundant and it can regulate metastatic onset by modulating neutrophil motility and response to cancer cells 13, 14. Previous studies showed that elevated CXCR4 expression in human CRC tissues positively correlated with lymph node metastasis, poor differentiation, high AJCC stage and poor prognosis 15, 16. Additionally, activation of the CXCR4 pathway promoted epithelial-mesenchymal transition, invasion and metastasis in human CRC 17. In line with these results, CXCR4 signaling blockade significantly reduced CXCL12-mediated CRC invasion and metastasis in preclinical models 18, 19. These works indicate that the CXCR4/CXCL12 axis plays an important role in CRC metastasis.

In this study, we aimed to characterize the biological function of transcription factor HOXB5 in CXCR4-mediated CRC metastasis. Our results revealed a positive feedback loop between HOXB5 and the CXCR4/CXCL12 axis which could be a promising therapeutic target for metastatic CRC.

## Materials and methods

### Patients and follow-up

Written informed consent was obtained from each patient, and ethical approval was obtained from the Ethics Committee of the Fourth Military Medical University. Cohort I included freshly sampled CRC tissues with healthy adjacent tissues collected between January 2005 and December 2007 from 334 adult patients who underwent surgery at Xijing Hospital of the Fourth Military Medical University (Xi'an, China). Cohort II included CRC tissue samples that were surgically resected from 390 adult CRC patients between January 2005 and December 2007 at the Tongji Hospital of Tongji Medical College (Wuhan, China). All patients were staged pathologically based on the American Joint Committee on Cancer (AJCC)/International Union against Cancer criteria. All patients were preoperative radiotherapy- and chemotherapy-naïve; however, those with stage II-IV disease received postoperative adjuvant chemotherapy. No patients were treated with postoperative radiotherapy. Primary tumor samples along with dissected regional lymph nodes were subjected to histomorphological analysis via hematoxylin-eosin (H&E) staining performed by the Department of Pathology of Xijing and Tongji Hospital. HOXB5 mRNA expression was assessed in 120 pairs of frozen fresh CRC tissues and peripheral nontumor tissues that were collected during surgical resection and frozen in liquid nitrogen.

The information collected during the follow-up period included the incidence of disease recurrence and the presence of distant metastasis as confirmed by imaging and procedural data (position emission tomography, ultrasonography, magnetic resonance imaging, computed tomography and endoscopy) or pathological data (biopsies and cytologic analysis). Overall survival time was defined as the period between surgical resection and death. The duration of disease-free survival was defined as the period between surgical resection and the emergence of either distant CRC metastasis or CRC recurrence, the occurrence of another noncolorectal cancer (with the exception of carcinoma *in situ* of the cervix and skin basal cell carcinoma) or death from any cause without documentation of a cancer-related event. Patients were followed up for a minimum of 8 years, with follow-up data collected via questionnaire letters and telephone inquiry; patient databases were updated every 3 months. Patient deaths were determined by a corroborative history from the family and verified by reviewing public records.

### Construction of tissue microarrays and immunohistochemical (IHC) staining

Tissue microarrays were constructed with the sampled human CRC tissues and their respective adjacent healthy tissues (Shanghai Biochip Co., Ltd. Shanghai, China). These microarrays were analyzed for HOXB5 (Abnova, PAB30143, 1/100), CXCR4 (Abcam, ab124824, 1/500), ITGB3 (Cell signaling technology, 13166S, 1/250) expression. All tissue microarrays were independently scored based on the degree of target protein expression and staining intensity by two pathologists who were blinded to the sample identities.

Sections of samples that were 4-μm thick were embedded in paraffin and used for IHC staining; paraffin-embedded sections were routinely processed. Briefly, slides were first incubated on a 60 °C heating panel for one hour before the paraffin was removed with xylene, and the sections were rehydrated using the gradient ethanol immersion technique. The sections were exposed to 3% (vol/vol) hydrogen peroxide for 12 minutes in methanol to quench endogenous peroxidase activity. The sections were then washed with PBS thrice for three minutes each time. Subsequently, the slides were placed into a microwave for half an hour while submerged in a 0.01 mol/L citrate buffer solution (pH 6.0). After the final PBS (pH 7.4, 0.01 mol/L) wash, the slides were incubated overnight with their primary antibodies diluted in PBS containing 1% (wt/vol) bovine serum albumin in a damp chamber at 4 °C. To produce negative controls, the same procedure was repeated on a separate set of slides, but preimmune mouse serum was used in place of the primary antibody. The following day, the slides were first washed thrice for 5 minutes each with PBS and exposed to a peroxidase-conjugated second antibody for half an hour (Dako, Carpinteria, CA, USA) at room temperature, followed by another three cycles of 5-minute PBS washes. Diaminobenzidine exposure for 2 minutes was used to visualize the reaction product, and images were captured with a DP70 digital camera-equipped light microscope (Olympus, Japan).

Analyses were performed by two independent observers who were blinded to the clinical outcome. The percentage of positive cells was scored on a scale of 0 to 4: 0 (negative), 1 (1%-25%), 2 (26%-50%), 3 (51%-75%), or 4 (76%-100%). The intensity of the immunostaining was scored on a scale of 0 to 3: 0 (negative), 1 (weak), 2 (medium) or 3 (strong). The product of the above two scores was used as the total immuno-activity score, which ranged from 0 to 12. The cut-off point for a 'high' score was a final score equal to or greater than 4, while scores from 0 to 3 were considered 'low'.

### *In vivo* metastatic model and bioluminescence imaging

Six-week-old BALB/C nude mice were cared for and maintained based on our institution's protocols for ethical animal care. The Committee on the Use of Live Animals in Teaching and Research (CULATR) of the Fourth Military Medical University approved all animal experiments. Mice were randomly assigned into experimental or control groups, blinding was not possible. In the tail vein injection-based *in vivo* metastasis assays, 10 mice in each group received tail vein injections of 1×10^6^ cells in 100 μL of phosphate-buffered saline (PBS). In the intrasplenic injection-based *in vivo* metastasis assays, the mice were first anesthetized by intraperitoneal injection (0.01 mL/mg) of a mixture of Zoletil (30 mg/kg) and Rompun (10 mg/kg). Spleens were exteriorized via a small left abdominal flank incision. A single intrasplenic injection of 2×10^6^ luciferase-labeled cells in 50 μL of Hank's balanced salt solution (HBSS) (Gibco) was administered with a 30-gauge needle. Gentle pressure was applied to the injection site with a cotton swab for one minute to staunch bleeding and to prevent leakage of tumor cells. Spleens were carefully reinserted into the abdominal cavity, and the wound was sutured using 6-0 black silk (10 mice per group). In the *in vivo* orthotopic CRC assays, the mice were first anesthetized by intraperitoneal injection (0.01 mL/mg) of a mixture of Zoletil (30 mg/kg) and Rompun (10 mg/kg). Ceca were exteriorized via a small left lower abdominal flank incision. 2×10^6^ luciferase-labeled cells in 50 μL Matrigel were injected into the cecum submucosa of nude mice with a 30-gauge needle. Gentle pressure was applied to the injection site with a cotton swab for one minute to prevent leakage of tumor cells. Ceca were carefully reinserted into the abdominal cavity, and the wound was sutured using 6-0 black silk (10 mice per group). Every week, the mice received intraperitoneal injections of 150 mg/kg of D-luciferin, and images were acquired 10 minutes after injection with an IVIS 100 Imaging System (Xenogen, Hopkinton, MA, USA). Each image was acquired within 2 minutes. The survival durations of the mice were monitored, and 9 weeks after tail vein and intrasplenic injections and 6 weeks after orthotopic cecal implantation, all mice were sacrificed for further histological examination for lung and liver metastases.

### The tumor metastasis RT^2^ profiler PCR array

Caco-2 cells were divided into two groups, namely Caco-2-control and Caco-2-HOXB5. RNA extraction, DNase treatment, and RNA cleanup were performed according to the manufacturer's protocol (Qiagen). The cDNA of each group was synthesized using the RT^2^ First Strand Kit (Qiagen). Gene expression profiling of Caco-2-control and Caco-2-HOXB5 cells was conducted using the Human Tumor Metastasis RT^2^ Profiler PCR Array, which represents 84 genes known to be involved in metastasis. The cDNA synthesis reaction was mixed with 2× RT^2^ qPCR SYBR Green Mastermix and ddH2O, and then dispensed to the PCR array 96-well plate (25 μL/well). A 2-step cycling program was performed using the Bio-Rad CFX96. Data normalization was done by correcting all Ct values based on the average Ct values of several housekeeping genes present on array. Each assay was conducted in triplicate.

### Chromatin immunoprecipitation (ChIP) assays

1 × 10^7^ CRC cells were used per ChIP analysis. Cells were cross-linked in 1% formaldehyde at 37 °C for 10 min. After washing with PBS, the cells were resuspended in 300 μl of lysis buffer. The DNA was sheared to small fragments by sonication. Sonicated chromatin was diluted to a final SDS concentration of 0.1% and aliquots were rotated with antibody O/N at 4 °C. The recovered supernatants were incubated with specific antibodies or an isotype control IgG for 2 hr in the presence of herring sperm DNA and Protein A/G Magnetic beads (Thermo Fisher). These antibodies are anti-HOXB5 (abcam, ab229345), and anti-ETS1 (CST, 14069S). The immunoprecipitated DNA was retrieved from the beads with 1% SDS and a 1.1 M NaHCO3 solution at 65 °C for 6 hr. The DNA was then purified using a PCR Purification Kit (Qiagen, USA). The primers are shown in Supplementary Table S4.

For ChIP assays of tissues, cells were first separated from six pairs of fresh frozen CRC tissues and normal colon tissues collected after surgical resection. In detail, surgically extracted tumor tissues were first washed by 1×cold, PBS, 5min, for three times and added to medium supplemented with antibiotic and antifungal agents. Use a clean razor blade to cut a pie of tissue (around 5 mm^3^) into small piece (typical 1mm^3^ or smaller). Then, digestion the tissues with DNase I (20 mg/mL; Sigma-Aldrich) and collagenase (1.5 mg/mL; Sigma-Aldrich) and placed on table concentrator, 37 °C, for 1 h. At the end of the hour, we filtered the dissociated cells through 100-μm-pore filters rinsed with fresh media. The 1×red cell lysis was added to the tissues and incubated for 5 minutes to lysis the red blood cell, followed by another rinse. The dissociated cells were crosslinked using 1% formaldehyde for 10 minutes at 37℃. After cell lysis, the DNA was fragmented by sonication. ChIP grade antibody anti-HOXB5 (abcam, ab229345), and anti-ETS1 (CST, 14069S), or IgG (negative control) was used to immunoprecipitated the fragment DNA. Then, qRT-PCR was used to amplify the corresponding binding site on the promoters.

### Agents

The CXCR4 inhibitor AMD3100 (HY-10046), ERK inhibitor SCH772984 (HY-50846), PI3K inhibitor LY294002 (HY-10108), mTOR inhibitor rapamycin (HY-10219), PKC inhibitor GO6983 (HY-13689) and PKA inhibitor H89 dihydrochloride (HY-15979A) were purchased from MedChemExpress (USA). Recombinant human CXCL12 protein was purchased from Bio-Techne (350-NS-010, R&D Systems, MN, USA). All the agents were used according to the manufacturer's instructions.

Detailed descriptions of all other materials and methods can be found in the online Supplementary Materials.

## Results

### Elevated expression of HOXB5 promotes CRC metastasis and indicates poor prognosis in human CRC

**Figure 1:**
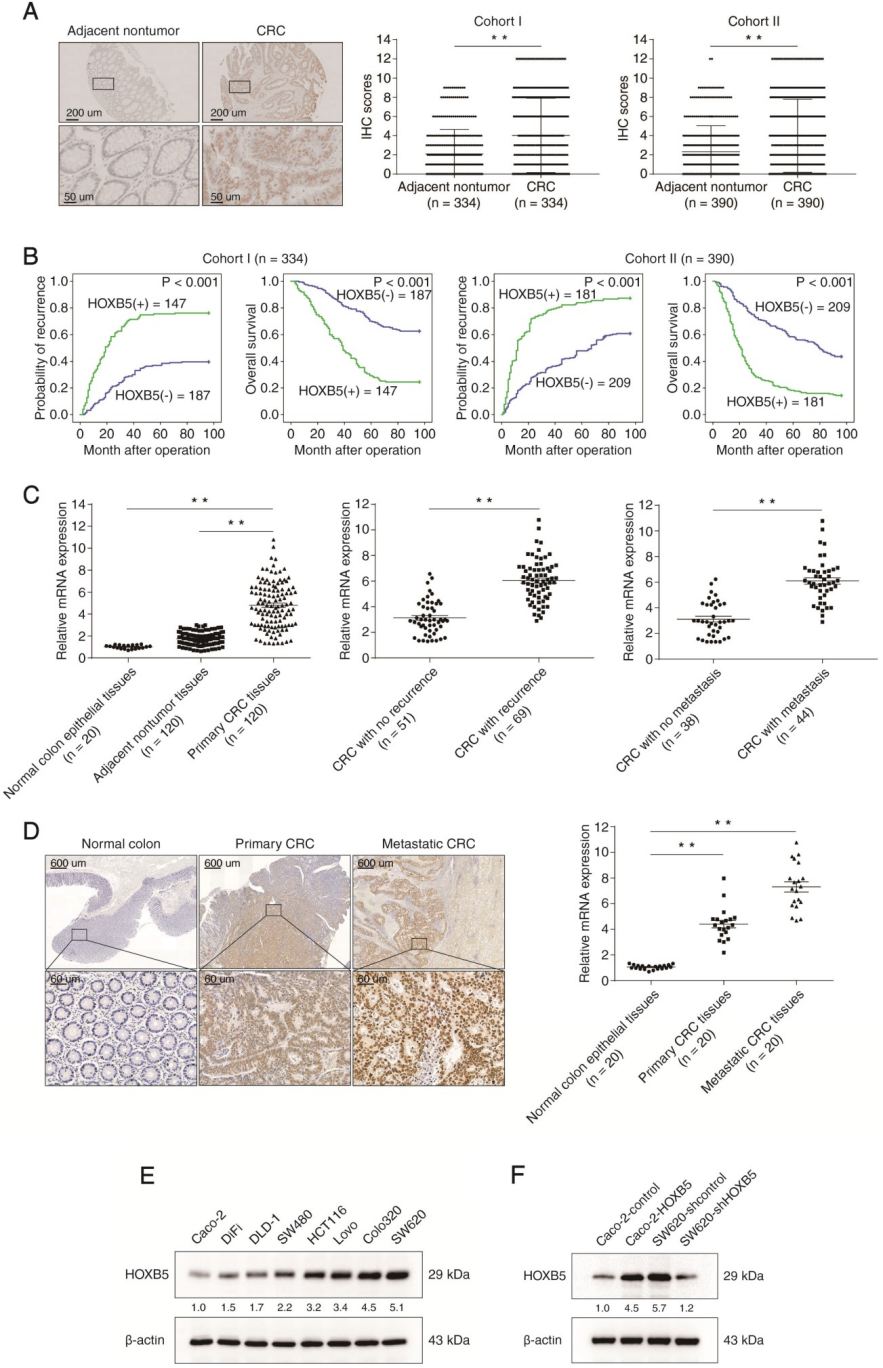

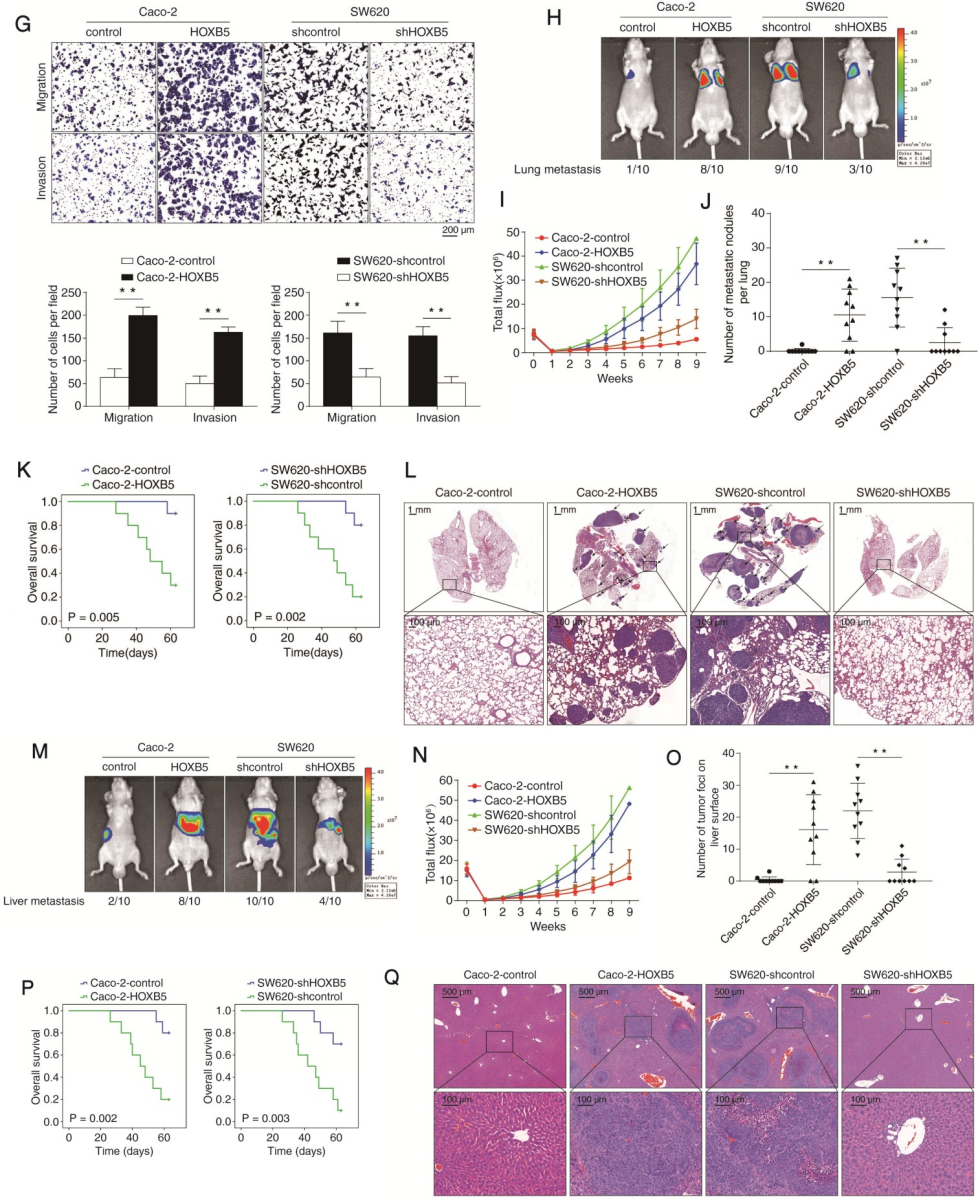
** Elevated expression of HOXB5 promotes CRC invasion and metastasis and predicts poor prognosis in human CRC. (A)** Left: representative images of IHC staining of HOXB5 in adjacent nontumor tissues and CRC tissues. The scale bars represent 200 µm (low magnification) and 50 µm (high magnification). Right: Evaluation of HOXB5 protein levels in CRC samples and paired adjacent nontumor samples by IHC scores in two independent cohorts. **(B)** Kaplan-Meier analysis of the correlation between HOXB5 expression and recurrence rates or overall survival times in two independent cohorts of CRC patients. **(C)** Left: real-time PCR analysis of *HOXB5* mRNA expression in normal colon epithelial tissues (n = 20) and 120 pairs of adjacent nontumor tissues and primary CRC tissues. Middle: relative mRNA expression of *HOXB5* in CRC samples from patients with recurrence (n = 69) or without recurrence (n = 51). Right: relative mRNA expression of *HOXB5* in CRC samples from patients with metastasis (n = 44) or without metastasis (n = 38). **(D)** The protein and mRNA levels of *HOXB5* were detected in 20 pairs of normal colon epithelial tissues, primary CRC tissues and matched metastatic CRC tissues by IHC and real-time PCR. Representative images of IHC staining (left) and real-time PCR analysis (right) of HOXB5 expression in normal colon epithelial tissues, primary CRC tissues and matched metastatic CRC tissues. The scale bars represent 600 µm (low magnification) and 60 µm (high magnification). **(E)** Western blotting analysis of HOXB5 protein expression in the established human CRC cell lines. **(F)** Western blotting analysis of HOXB5 protein expression in the indicated CRC cell lines after lentivirus transfection. **(G)** Transwell assay analysis of the migration and invasion abilities of the indicated CRC cell lines. **(H-L)** *In vivo* lung metastatic assays. Four stable cell lines were injected into the tail veins of nude mice (n = 10 mice per group). (H) Representative bioluminescent images of the different groups at 9 weeks after implantation and the incidence of lung metastasis are shown. (I) The intensity of recorded bioluminescence signals for 9 consecutive weeks. (J) The number of lung metastatic nodules. (K) Overall survival of the nude mice in the different groups. (L) Representative images of H&E staining of lung tissues from the different groups were shown. The scale bars represent 1 mm (low magnification) and 100 µm (high magnification). **(M-Q)** *In vivo* liver metastatic assays. Four stable cell lines were injected into the spleens of nude mice (n = 10 mice per group). (M) Representative bioluminescent images of the different groups at 9 weeks after implantation and the incidence of liver metastasis were shown. (N) The intensity of recorded bioluminescence signals for 9 consecutive weeks. (O) The number of liver metastatic nodules. (P) Overall survival of the nude mice in the different groups. (Q) Representative images of H&E staining of liver tissues from the different groups were shown. The scale bars represent 500 µm (low magnification) and 100 µm (high magnification). All the data are shown as the mean ± s.d. *P < 0.05 **P ˂ 0.01.

To investigate the potential role of HOXB5 in CRC, we used immunohistochemical (IHC) staining to examine its expression in human CRC tissues from two independent cohorts (cohort Ⅰ, n = 334; cohort II, n = 390). We found that the protein levels of HOXB5 in CRC tissues were significantly higher than in adjacent non-tumor tissues (Figure 1A). In both cohorts, elevated expression of HOXB5 positively correlated with lymph node metastasis, distant metastasis, poor tumor differentiation and advanced AJCC stage (Table 1). Compared with HOXB5-negative CRC patients, HOXB5-positive patients possessed higher recurrence risks and shorter overall survival times (Figure 1B). Next, the mRNA levels of *HOXB5* were analyzed by real-time PCR in 20 normal colon epithelial tissues and 120 paired adjacent non-tumor tissues and primary CRC tissues. The mRNA levels of *HOXB5* in primary CRC tissues were significantly higher than in adjacent nontumor tissues and normal colon epithelial tissues (Figure 1C). Additionally, the mRNA levels of *HOXB5* in primary CRC tissues from patients with recurrence or metastasis were dramatically elevated than those in primary CRC tissues from patients without recurrence or metastasis (Figure 1C). Next, we examined the protein and mRNA expression levels of HOXB5 in 20 pairs of normal colon epithelial tissues, primary CRC tissues and matched metastatic CRC tissues with IHC and real-time PCR. Similarly, the mRNA and protein levels of HOXB5 are markedly higher in metastatic CRC tissues than in primary CRC tissues and normal colon epithelial tissues (Figure 1D).

To explore the function of HOXB5 in CRC metastasis, we profiled its protein level in a panel of CRC cell lines. HOXB5 expression was higher in CRC cells with high metastatic capability (LoVo, Colo320, and SW620) than in CRC cells with low metastatic capability (Caco-2, DiFi, DLD-1, SW480, and HCT116) (Figure 1E). HOXB5-low Caco-2 and HOXB5-high SW620 cells were selected to generate two stable cell lines, Caco-2-HOXB5 and SW620-shHOXB5 (Figure 1F). Results of transwell assays showed that the upregulation of HOXB5 promoted the migration and invasion of Caco-2 cells, whereas knockdown of HOXB5 weakened the migratory and invasive abilities of SW620 cells (Figure 1G).

*In vivo* lung metastatic models showed that HOXB5 overexpression increased lung metastasis burden and the intensity of bioluminescence imaging (BLI) signals, while shortened the overall survival time in the Caco-2-HOXB5 group. Contrastingly, HOXB5 knockdown reduced lung metastasis burden and the intensity of BLI signals, while prolonged the overall survival time of the SW620-shHOXB5 group (Figure 1H-L). To validate these results, we conducted the intrasplenic injection-based *in vivo* liver metastatic assay. Indeed, HOXB5 overexpression increased the liver metastasis burden and led to shorter overall survival time in the Caco-2-HOXB5 group. Conversely, HOXB5 knockdown decreased the liver metastasis burden and the BLI signals intensity while extending the overall survival time of the SW620-shHOXB5 group (Figure 1M-Q). Compared with the widely used xenograft mouse models, orthotopic tumor mouse models were considered to be a better system to study tissue-site specific metastasis. Therefore, we also established orthotopic CRC models to explore the role of HOXB5 in CRC metastasis. Consistently, the results derived from orthotopic CRC models also showed that HOXB5 overexpression significantly promoted CRC metastasis* in vivo* (Supplementary Figure S1A-G). Adding together, these results provided compelling evidence for the driver function of HOXB5 in CRC metastasis.

To investigate the role of HOXB5 in CRC cell proliferation and tumor growth, we performed Cell Counting Kit-8 (CCK8) and colony formation assays to measure changes of cell proliferation after the manipulation of HOXB5 expression level. Overexpression of exogenous HOXB5 significantly promoted the proliferation of Caco-2 cells with low level of endogenous HOXB5, whereas depletion of endogenous HOXB5 markedly decreased the proliferation of SW620 cells with high basal level of HOXB5 (Supplementary Figure S2A-B). Next, we set out to study the oncogenic role of HOXB5 in CRC using *in vivo* tumorigenicity assays. Compared with the isogenic controls, exogenous HOXB5 expression in Caco-2 cells significantly increased the growth of subcutaneous tumors in mice, while depletion of endogenous HOXB5 in SW620 cells dramatically decreased tumor growth (Supplementary Figure S2C-E). Collectively, these data suggested that HOXB5 promoted CRC cell proliferation both *in vitro* and *in vivo*.

### Metastasis-related genes CXCR4 and ITGB3 are downstream targets of HOXB5

**Figure 2:**
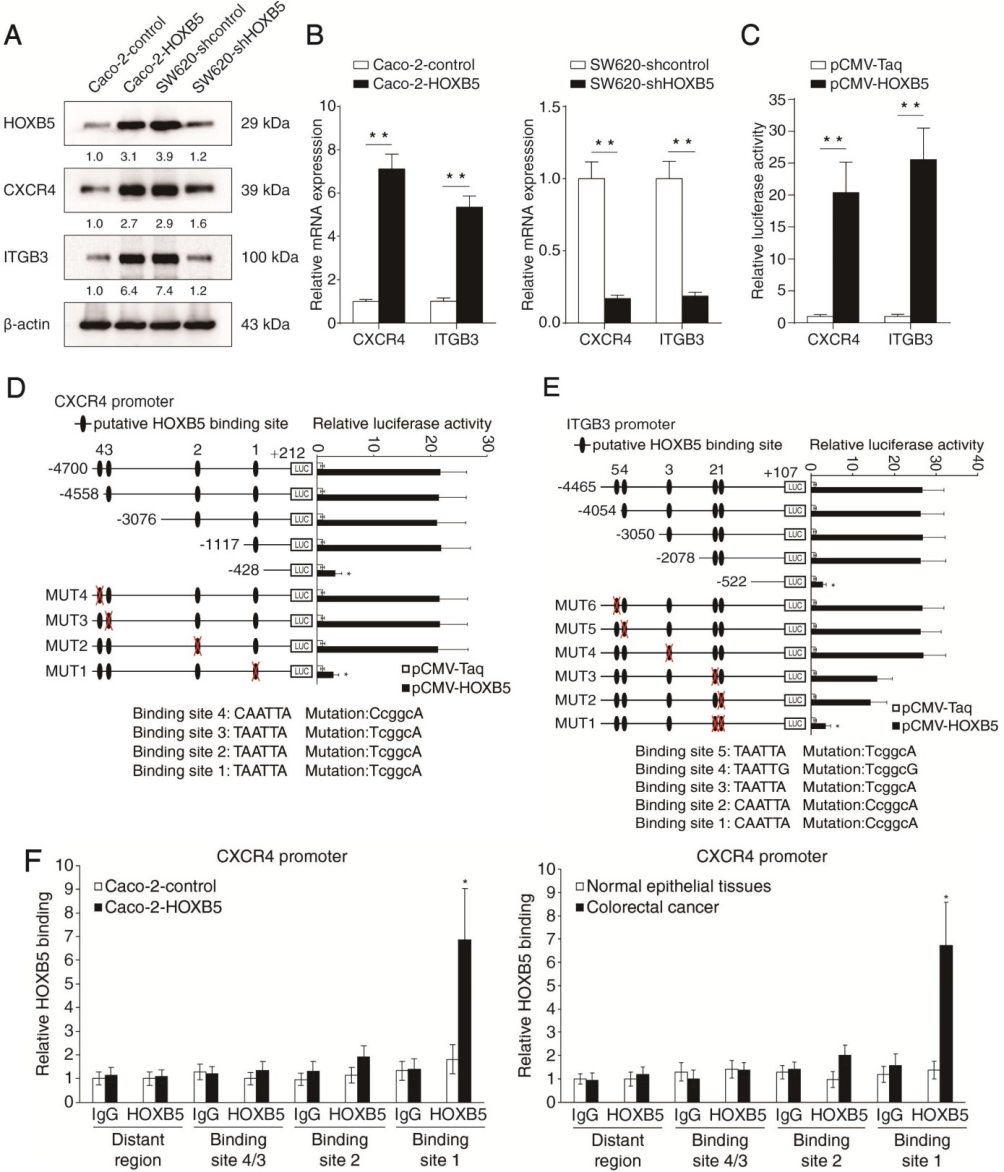

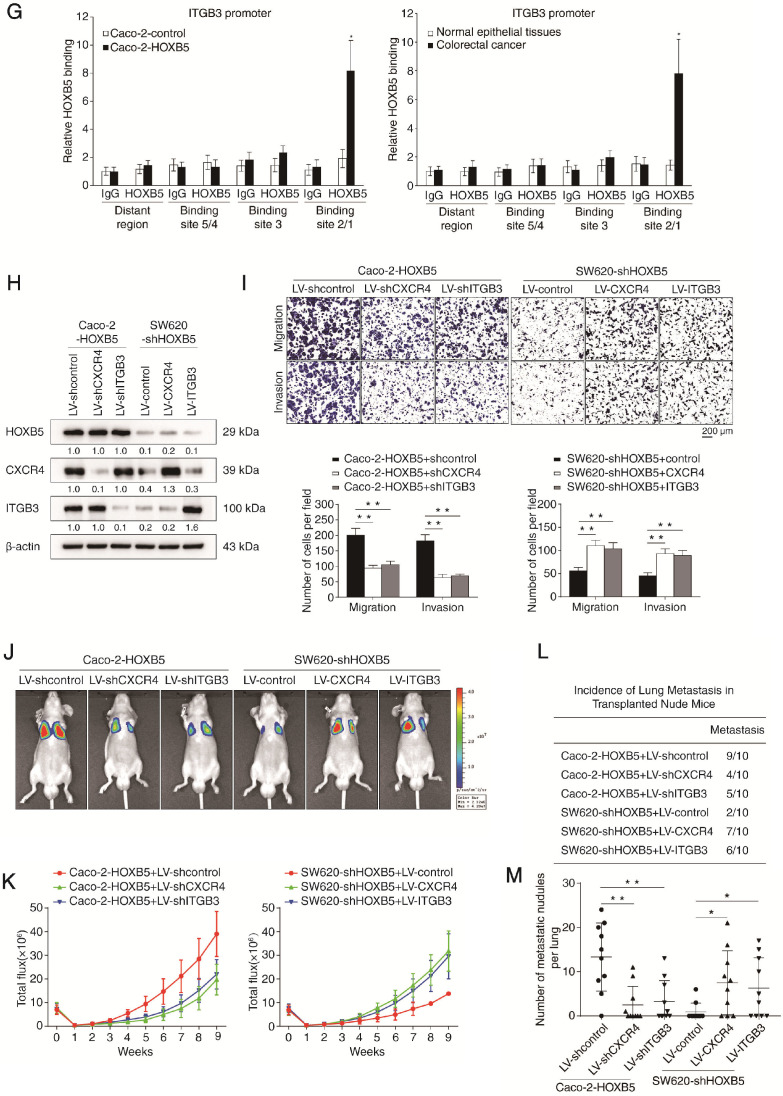

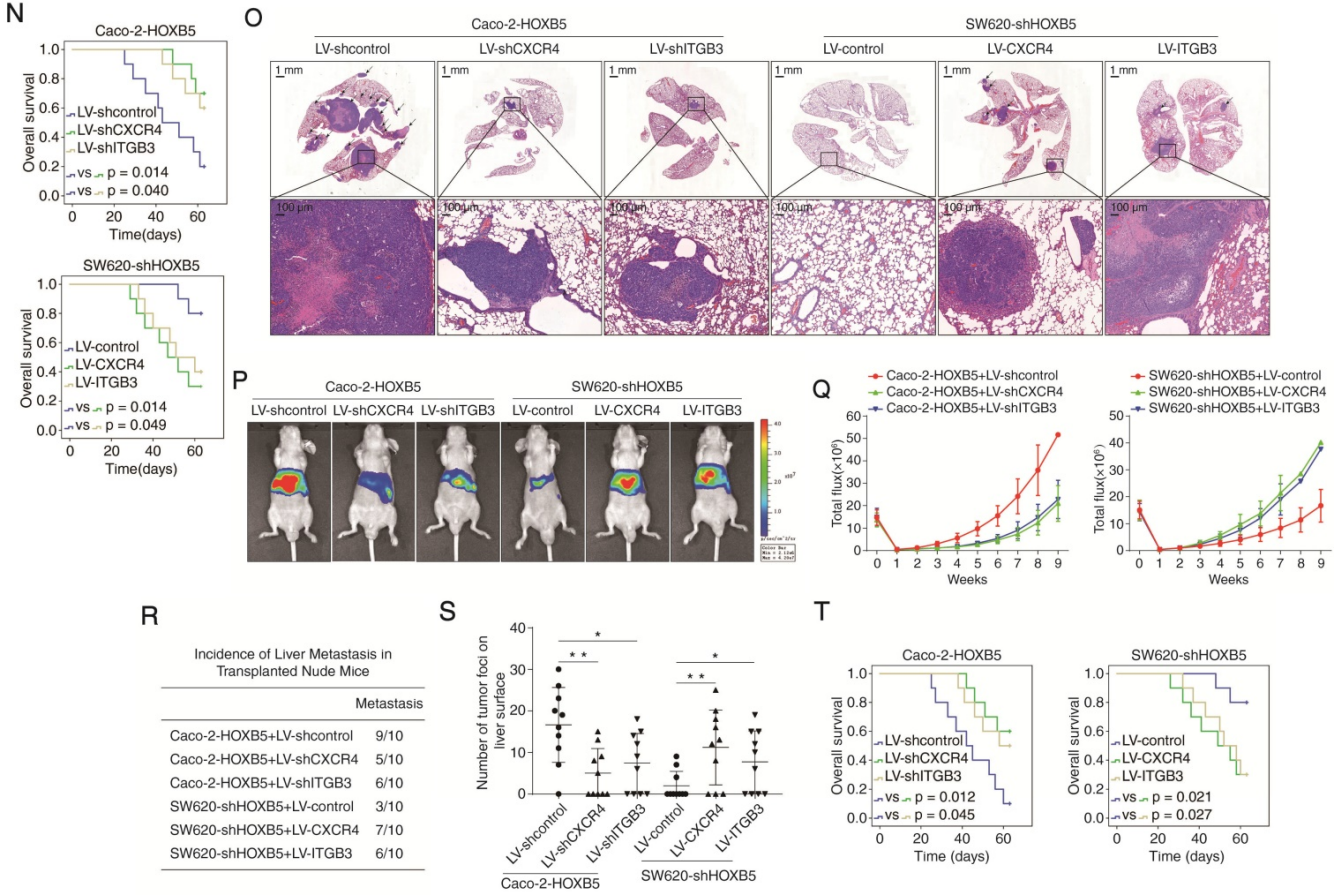

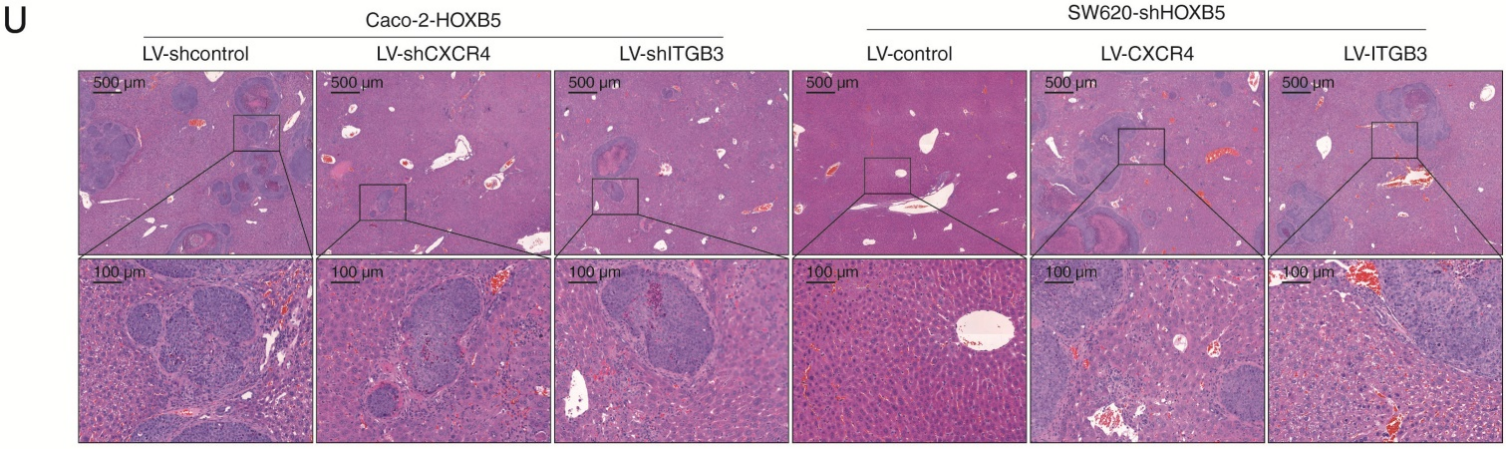
** HOXB5 promotes CRC metastasis through upregulating CXCR4 and ITGB3 expression. (A)** Western blotting analysis of CXCR4 and ITGB3 expression in the indicated CRC cell lines after manipulation of HOXB5 expression via lentivirus transfection. **(B)** Real-time PCR analysis of *CXCR4* and *ITGB3* mRNA expression in the indicated CRC cell lines. **(C)** HOXB5 transactivates *CXCR4* and *ITGB3* promoters. Caco-2 cells were co-transfected with pCMV-HOXB5 and *CXCR4* or *ITGB3* promoter luciferase constructs, then promoter activities were examined by a luciferase reporter assay. **(D-E)** Deletion and selective mutation analysis identified HOXB5-responsive regions in the *CXCR4* (D) or *ITGB3* (E) promoter. PGL3-luciferase reporter plasmids containing serially truncated or mutated *CXCR4* or *ITGB3* promoter constructs were co-transfected with pCMV-HOXB5 into Caco-2 cells, and relative luciferase activities were detected. **(F-G)** A CHIP assay indicated that the direct binding of HOXB5 to the *CXCR4* promoter (F) and *ITGB3* promoter (G) in both Caco-2-HOXB5 cells and human primary CRC tissues. Cells were cross-linked and the chromatin was immunoprecipitated by anti-HOXB5 or control antibody, respectively. Real-time PCR was then carried out to quantitate the immunoprecipitated products by using primers within the *CXCR4* or *ITGB3* promoter. **(H)** Western blotting analysis of CXCR4 and ITGB3 expression in the Caco-2 and SW620 cells after lentivirus transfection. **(I)** Transwell assay analysis demonstrated that downregulation of CXCR4 and ITGB3 inhibited the migration and invasion capacities of Caco-2-HOXB5 cells, and upregulation of CXCR4 or ITGB3 promoted the migratory and invasive potentials of SW620-shHOXB5 cells. **(J-O)** *In vivo* lung metastatic assays. Indicated cell lines were injected into the tail veins of nude mice (n = 10 mice per group). (J) Representative bioluminescent images of the different groups at 9 weeks after implantation were shown. (K) The intensity of recorded bioluminescence signals for 9 consecutive weeks of the different groups was presented. (L) Incidence of lung metastasis in the different groups. (M) The number of lung metastatic nodules in the different groups. (N) Overall survival time of nude mice in the different groups. (O) Representative images of H&E staining of lung tissues from the different groups were shown. The scale bars represent 1 mm (low magnification) and 100 µm (high magnification). **(P-U)** *In vivo* liver metastatic assays. Indicated cell lines were injected into the spleens of nude mice (n = 10 mice per group). (P) Representative bioluminescent images of the different groups at 9 weeks after implantation were shown. (Q) The bioluminescence signals of the different groups were recorded for 9 consecutive weeks after cell implantation. (R) Incidence of liver metastasis in the different groups. (S) The number of metastatic nodules in the liver was calculated. (T) Overall survival time of nude mice in the different groups. (U) Representative images of H&E staining of liver tissues from the different groups were shown. The scale bars represent 500 µm (low magnification) and 100 µm (high magnification). All the data are shown as the mean ± s.d. *P < 0.05 **P ˂ 0.01.

To explore the mechanism by which HOXB5 promotes CRC metastasis, we profiled the expression of a series of metastasis-related genes in Caco-2 and the isogenic Caco-2-HOXB5 cells using a Tumor Metastasis RT^2^ Profiler PCR array. HOXB5 upregulation resulted in expression changes of multiple metastasis-related genes, such as *CXCR4*, *ITGB3*, *MAT1*, *ETV4, MMP2, MMP7* and* MMP13* (Supplementary Table S1, Supplementary Figure S3). Among these genes, *CXCR4* and *ITGB3* came to our attention due to their critical roles in CRC metastasis 20, 21. Using real-time PCR and Western blotting, we validated that the mRNA and protein levels of CXCR4 and ITGB3 were significantly upregulated upon the overexpression of HOXB5 in Caco-2 cells, whereas knockdown of *HOXB5* in SW620 cells markedly decreased the mRNA and protein levels of CXCR4 and ITGB3 (Figure 2A-B). Results of the luciferase reporter assay also showed that exogenous HOXB5 enhanced the expression of reporter genes driven by the *CXCR4* and *ITGB3* promoters (Figure 2C).

Through *in silico* sequence analysis, we identified multiple putative HOXB5-binding motifs in the promoters of *CXCR4* (n = 4) and *ITGB3* (n = 5). To explore the functions of these motifs in transcription regulation, we constructed a series of reporter genes driven by truncated or mutated *CXCR4* promoter sequences. Results of the luciferase reporter assay demonstrated that deletion of the region between -1117 to -428 base pairs largely abolished the increased activity of the *CXCR4* promoter modulated by HOXB5 overexpression. Consistently, mutation of the putative HOXB5-binding motif 1 located in this region significantly impaired HOXB5-induced *CXCR4* promoter activity (Figure 2D). The same approach was used to characterize HOXB5-dependent *cis* elements in the *ITGB3* promoter. Through deletion mapping and site-directed mutagenesis, we found that the putative HOXB5-binding motif 1 and 2 located in the fragment between -2078 to -522 base pairs were required for HOXB5-mediated *ITGB3* transactivation (Figure 2E). Furthermore, we validated the binding of endogenous HOXB5 to the motifs described above in CRC cells and human CRC tissues with chromatin immunoprecipitation (ChIP) assay (Figure 2F-G). These results confirmed that *CXCR4* and *ITGB3* were direct transcriptional targets of HOXB5.

### HOXB5 promotes CRC metastasis through upregulating CXCR4 and ITGB3 expression

To determine whether CXCR4 and ITGB3 are required for HOXB5-mediated CRC invasion and metastasis, we applied *CXCR4* and *ITGB3* knockdown in Caco-2 cells expressing exogenous HOXB5 (Caco-2-HOXB5) and overexpression of CXCR4 and ITGB3 in HOXB5-depleted SW620 cells (SW620-shHOXB5) (Figure 2H). Results of transwell assays showed that *CXCR4* and *ITGB3* knockdown decreased the migration and invasion capacities of the Caco-2-HOXB5 cells. Reciprocally, CXCR4 and ITGB3 overexpression rescued the migratory and invasive defects of the SW620-shHOXB5 cells (Figure 2I).

To visualize cancer metastasis in mouse models, we integrated bioluminescence imaging (BLI) in our *in vivo* lung metastatic assays. Our results showed that *CXCR4* and *ITGB3* knockdown in Caco-2-HOXB5 cells decreased the intensity of BLI signals, the incidence of lung metastasis and the number of lung metastatic nodules. Consistently, *CXCR4* and *ITGB3* knockdown also prolonged the overall survival time of the nude mice. On the contrary, overexpression of CXCR4 and ITGB3 rescued various phenotypes of HOXB5 depletion in SW620 cells including reduction of lung metastasis burden, decreased BLI signals intensity and shortened overall survival time of the nude mice (Figure 2J-O). We also saw similar results in our *in vivo* liver metastatic assays. *CXCR4* and *ITGB3* knockdown in the Caco-2-HOXB5 cells decreased the BLI signals intensity, the incidence of liver metastasis and the number of liver metastatic nodules while extending the overall survival time of the nude mice. In contrast, overexpression of CXCR4 and ITGB3 in the SW620-shHOXB5 cells promoted liver metastasis and shortened the overall survival time of the xenograft nude mice (Figure 2P-U). All these findings suggested that HOXB5 facilitated CRC metastasis through the upregulation of CXCR4 and ITGB3 expression.

### HOXB5 expression positively correlated with CXCR4 and ITGB3 expression in human CRC tissues

**Figure 3:**
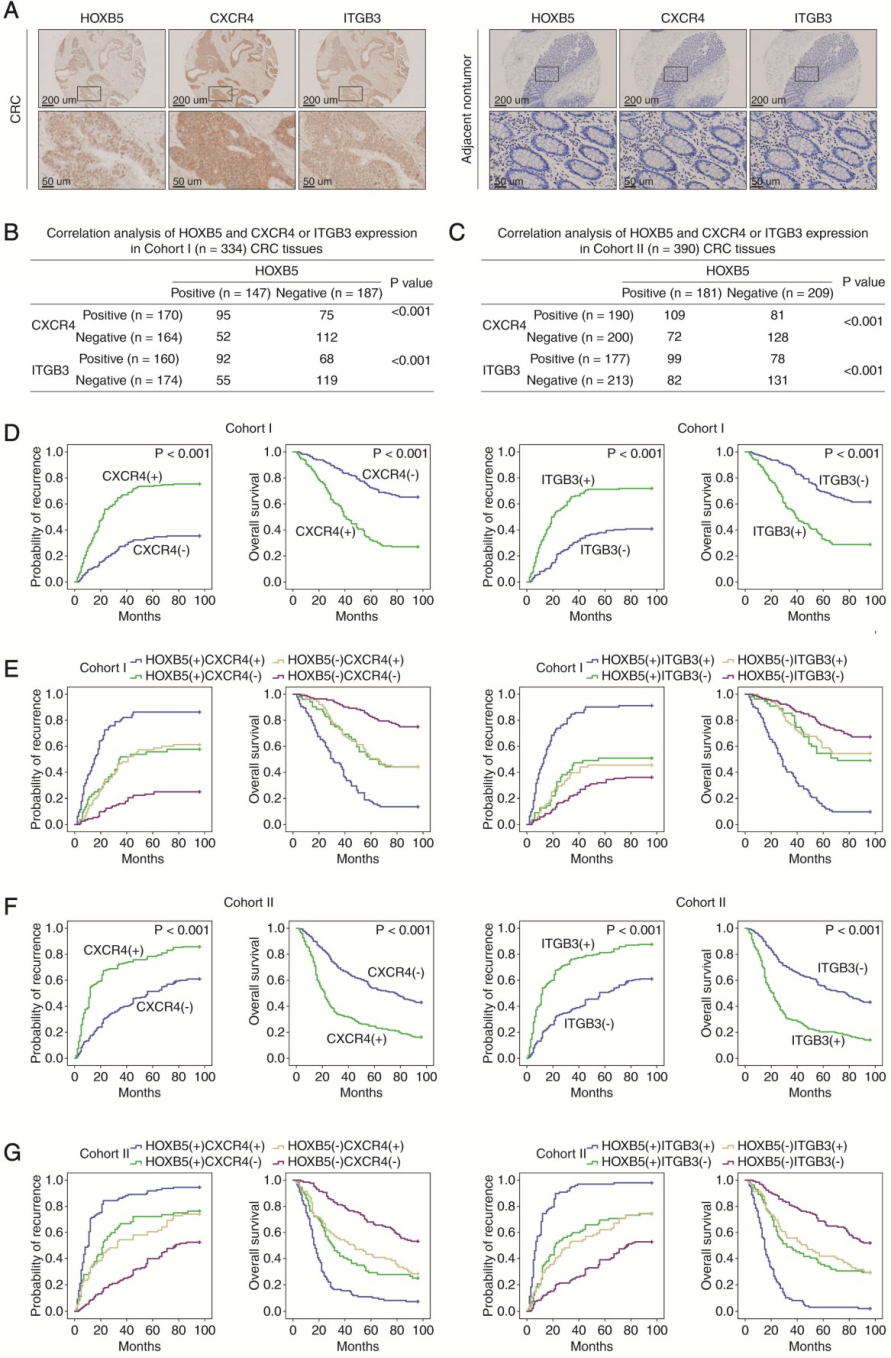
** HOXB5 expression is positively correlated with CXCR4 and ITGB3 expression in human CRC tissues. (A)** Representative images of IHC staining of HOXB5, CXCR4 and ITGB3 expression in CRC tissues and adjacent nontumor tissues were shown. The scale bars represent 200 µm (low magnification) and 50 µm (high magnification). **(B-C)** Correlation analysis of HOXB5 expression and CXCR4 or ITGB3 expression in CRC tissues in cohort I (B) and cohort II (C). **(D)** Kaplan-Meier's analysis of the correlation between CXCR4 expression (left) or ITGB3 expression (right) and recurrence or overall survival of CRC patients in cohort I. **(E)** Kaplan-Meier's analysis of the correlation between HOXB5/CXCR4 co-expression (left) or HOXB5/ITGB3 co-expression (right) and recurrence or overall survival of CRC patients in cohort I. **(F)** Kaplan-Meier's analysis of the correlation between CXCR4 expression (left) or ITGB3 expression (right) and recurrence or overall survival of CRC patients in cohort II. **(G)** Kaplan-Meier's analysis of the correlation between HOXB5/CXCR4 co-expression (left) or HOXB5/ITGB3 co-expression (right) and recurrence or overall survival of CRC patients in cohort II.

The positive correlation between HOXB5 expression and its target genes CXCR4 and ITGB3 in human CRC cell lines led us to explore whether it also existed in primary tumor tissues. To address this question, we used IHC staining to analyze the expression of HOXB5, CXCR4 and ITGB3 in primary tumor tissues and adjacent non-tumor tissues from two independent CRC patient cohorts (Figure 3A). In both cohorts, HOXB5 expression positively correlated with CXCR4 and ITGB3 expression (Figure 3B-C), and elevated expression of CXCR4 and ITGB3 positively associated with lymph node metastasis, distant metastasis and advanced AJCC stage (Supplementary Table S2-S3). Importantly, patients with positive CXCR4 or ITGB3 expression exhibited higher recurrence rates and shorter overall survival times than patients with negative CXCR4 or ITGB3 expression (Figure 3D, 3F). Kaplan-Meier analysis revealed that the subgroups of patients with co-expression of HOXB5/CXCR4 or HOXB5/ITGB3 had the highest recurrence rates and shortest overall survival times in our cohorts (Figure 3E, 3G).

### CXCL12 upregulates HOXB5 expression through the CXCR4-ERK1/2-ETS1 signaling pathway

Chemokine CXCL12 plays an important role in the communication between tumor cells and their surrounding microenvironment. The binding of CXCL12 to its cognate receptor CXCR4 triggers divergent downstream signaling pathways, which in turn regulates multiple biological processes such as chemotaxis, cell survival, proliferation, migration and gene transcription 13, 22. Aberrant transactivation of oncogenes by the CXCL12-CXCR4 signaling pathway has been reported in multiple human cancer types 23-26. The CXCL12-CXCR4 signaling accelerates CRC progression by promoting EMT, cell migration and metastasis 17, 27. Given the critical roles of the CXCL12/CXCR4 axis and HOXB5 in CRC metastasis, we set out to explore whether the CXCL12-CXCR4 axis regulates HOXB5 expression in the context of CRC.

**Figure 4:**
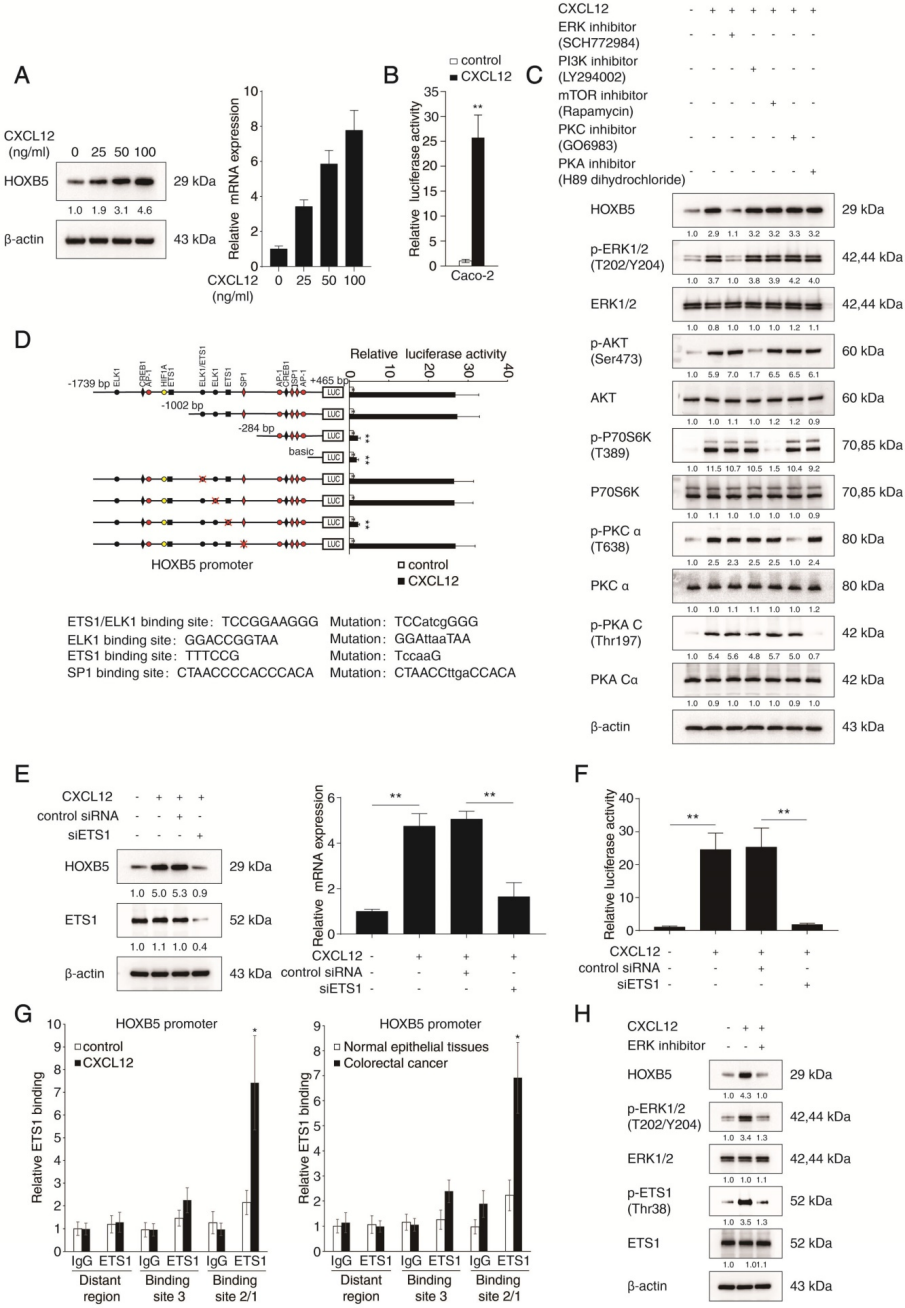
** CXCL12 upregulates HOXB5 expression through the CXCR4-ERK1/2-ETS1 signaling pathway. (A)** Caco-2 cells were treated with CXCL12 of gradient concentrations for 24 hours, then *HOXB5* protein and mRNA expression was detected by western blotting and real-time PCR. **(B)** Relative luciferase activities were determined in Caco-2 cells transfected with *HOXB5* promoter luciferase reporter after CXCL12 incubation (100 ng/ml) for 24 hours. **(C)** Caco-2 cells were cultured with specific inhibitors of ERK, PI3K, mTOR, PKA or PKC and then treated with or without CXCL12 (100 ng/ml, 24 hours). The protein levels of HOXB5 and of total and phosphorylated ERK, AKT, P70S6K, PKC and PKA were detected by Western blotting. **(D)** Deletion and selective mutation analysis showed that an ETS1 binding region within the *HOXB5* promoter was responsible for CXCL12-mediated HOXB5 expression. Caco-2 cells were transfected with serially truncated or mutated *HOXB5* promoter luciferase constructs and incubated with CXCL12 (100 ng/ml) for 24 hours, then the luciferase activities were determined. **(E-F)** Knockdown of ETS1 impaired CXCL12-mediated HOXB5 expression. Caco-2 cells were transfected with ETS1 siRNA or control siRNA and then treated with or without CXCL12 (100 ng/ml, 24 hours). HOXB5 expression was measured by Western blotting and real-time PCR (E). The activity of *HOXB5* promoter was examined by luciferase reporter assays (F). **(G)** A CHIP assay indicated the direct binding of ETS1 to the *HOXB5* promoter in Caco-2 cells and human primary CRC tissues. **(H)** Caco-2 cells were incubated with or without ERK inhibitor and then treated with or without CXCL12 (100 ng/ml, 24 hours). Western blotting assays were conducted to detect the protein levels of HOXB5, ERK, ETS1, phosphorylated ERK and phosphorylated ETS1.

To investigate the potential regulatory loop between the CXCL12-CXCR4 axis and HOXB5, Caco-2 cells with low basal level of HOXB5 were treated with CXCL12 for 24 hours. CXCL12 treatment significantly upregulated HOXB5 expression in a dose-dependent manner (Figure 4A). A luciferase reporter assay demonstrated that the transcriptional activity of the *HOXB5* promoter was markedly induced by CXCL12 treatment (Figure 4B). The CXCL12-CXCR4 axis is known to activate several intracellular signaling pathways, including extracellular regulated protein kinase (ERK), phosphoinositide 3-kinase (PI3K), mechanistic target of rapamycin kinase (mTOR), protein kinase C (PKC) and protein kinase A (PKA) 22. To identify which signaling pathway is involved in CXCL12-dependent HOXB5 induction, Caco-2 cells were treated with inhibitors targeting different signaling pathways, including ERK inhibitor SCH772984, PI3K inhibitor LY294002, mTOR inhibitor rapamycin, PKC inhibitor GO6983 and PKA inhibitor H89 dihydrochloride. The treatment of ERK inhibitor (SCH772984) significantly impaired CXCL12-dependent HOXB5 induction in Caco-2 cells, whereas other inhibitors had no such effect (Figure 4C). This result suggested that the ERK signaling is required for CXCL12-dependent HOXB5 induction.

To characterize the cis-regulatory elements within the *HOXB5* promoter that mediate CXCL12-dependent HOXB5 induction, we analyzed the *HOXB5* promoter sequence and discovered several putative binding sites of transcription factors involved in the ERK signaling. Next we generated a series of luciferase reporter constructs driven by truncated or mutated *HOXB5* promoters and transfected them into Caco-2 cells. Interestingly, deletion of the region from -1002bp to -284bp within the *HOXB5* promoter significantly reduced CXCL12-dependent *HOXB5* promoter activity in Caco-2 cells (Figure 4D), suggesting that this region was essential for CXCL12-dependent HOXB5 induction. Furthermore, disruption of a putative ETS1 binding site in this region dramatically reduced CXCL12-dependent *HOXB5* promoter activity. In contrast, mutations of other transcription factor binding sites (SP1, ELK1, and ELK1/ETS1 binding sites) in this region did not affect the *HOXB5* promoter activity (Figure 4D). Consistently, *ETS1* knockdown significantly reduced *HOXB5* induction mediated by CXCL12 treatment and CXCL12-dependent *HOXB5* promoter activity in Caco-2 cells (Figure 4E-F), indicating that ETS1 was indispensable for CXCL12-dependent HOXB5 transactivation. Results of the ChIP assays confirmed the binding of ETS1 to the *HOXB5* promoter in both CXCL12-treated CRC cells and human CRC tissues (Figure 4G). Importantly, ERK inhibitor (SCH772984) treatment led to the simultaneous decrease of phosphorylated ETS1 and HOXB5 in CXCL12-treated Caco-2 cells (Figure 4H), suggesting that the ERK/ETS1 signaling pathway was required for CXCL12-dependent HOXB5 induction. These findings demonstrated that CXCL12 upregulated HOXB5 expression through the CXCR4-ERK1/2-ETS1 signaling cascade.

### HOXB5 is essential for CXCL12-mediated CRC invasion and metastasis

**Figure 5:**
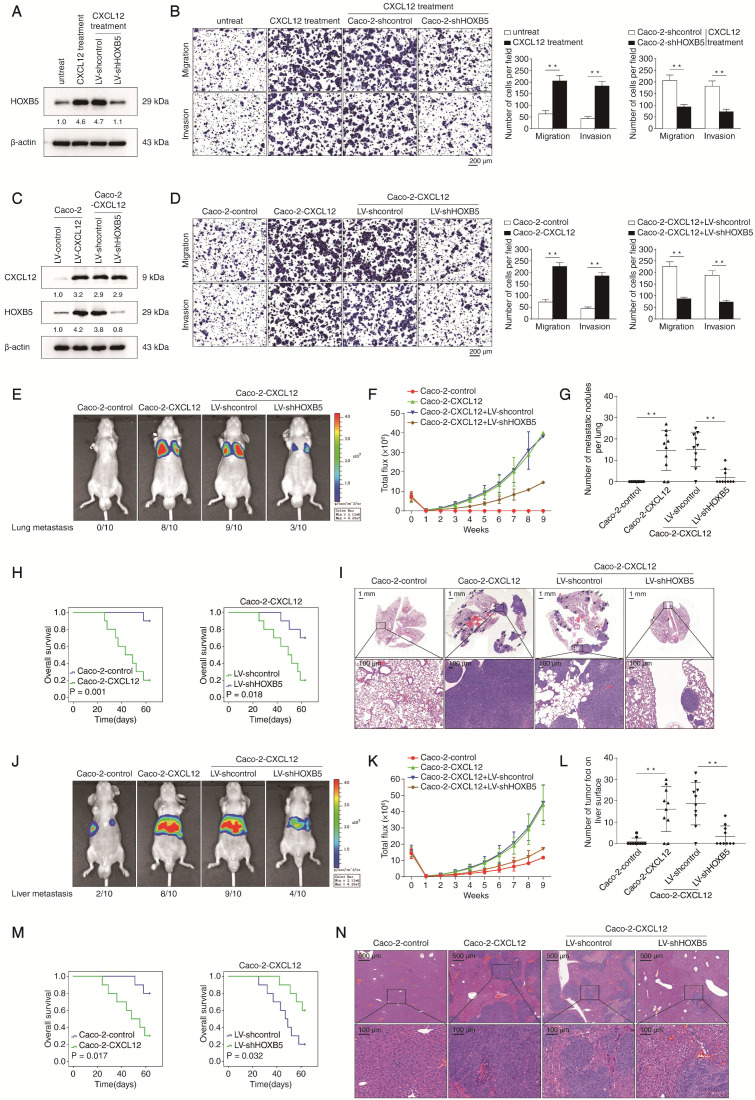
** HOXB5 is essential for CXCL12-mediated CRC metastasis. (A)** Caco-2 cells were transfected with LV-shcontrol or LV-shHOXB5 lentiviral vectors and then treated with or without CXCL12 (100 ng/ml, 24 hours). Next, the HOXB5 protein expression was measured by Western blotting. **(B)** Transwell assays demonstrated that CXCL12 treatment significantly increased the migration and invasion of Caco-2 cells while HOXB5 knockdown reduced CXCL12-enhanced migration and invasion abilities in Caco-2 cells. **(C)** Caco-2 cells were transfected with lentiviral vectors to construct CXCL12-overexpressing Caco-2 cells (Caco-2-CXCL12), and HOXB5 expression was further knockdown via lentiviral transfection in Caco-2-CXCL12 cells. CXCL12 and HOXB5 expression in the indicated cell lines was examined by Western blotting. **(D)** Transwell assays showed that CXCL12 overexpression markedly promoted the migration and invasion of Caco-2 cells, and HOXB5 knockdown significantly reduced the enhanced migratory and invasive potentials in Caco-2-CXCL12 cells. **(E-I)** *In vivo* lung metastatic assays showed that HOXB5 knockdown inhibited CXCL12-mediated CRC metastasis. Four stable cell lines were injected into the tail veins of nude mice (n = 10 mice per group). (E) Representative bioluminescent images of the different groups at 9 weeks after implantation and the incidence of lung metastasis were shown. (F) The intensity of bioluminescence signals for 9 consecutive weeks of the different groups. (G) The number of lung metastatic foci in the different groups. (H) Overall survival time of nude mice in the different groups. (I) Representative images of H&E staining of lung tissues from the different groups. The scale bars represent 1 mm (low magnification) and 100 µm (high magnification). **(J-N)*** In vivo* liver metastatic assays. Four stable cell lines were injected into the spleens of nude mice (n = 10 mice per group). (J) Representative bioluminescent images of the different groups at 9 weeks after implantation and the incidence of liver metastasis. (K) The bioluminescence signals of the different groups were recorded for 9 consecutive weeks after cell implantation. (L) The number of metastatic nodules in the liver of the four groups. (M) Overall survival time of nude mice in the different groups. (N) Representative images of H&E staining of liver tissues from the different groups. The scale bars represent 500 µm (low magnification) and 100 µm (high magnification). All the data are shown as the mean ± s.d. *P < 0.05, **P ˂ 0.01.

To explore the potential role of HOXB5 in CXCL12-mediated CRC metastasis, we performed *HOXB5* knockdown in Caco-2 cells and then treated the cells with 100 ng/ml CXCL12 for 24 hours (Fig 5A). CXCL12 treatment significantly promoted the migration and invasion of Caco-2 cells which was reversed by *HOXB5* knockdown (Figure 5B). Next, we established a CXCL12-overexpressing Caco-2 cell line (Caco-2-CXCL12) and depleted *HOXB5* expression in this cell line with shRNA knockdown. Expression of exogenous CXCL12 and depletion of endogenous HOXB5 in the indicated cell lines were validated by Western blotting assay (Figure 5C). Overexpression of CXCL12 significantly increased the migration and invasion of Caco-2 cells which was dramatically abolished by *HOXB5* knockdown (Figure 5D).

Results of* in vivo* lung metastatic assays showed that overexpression of CXCL12 in Caco-2 cells increased the intensity of BLI signals, the incidence of lung metastasis and the number of lung metastatic nodules, which correlated with shortened overall survival times of the nude mice. These CXCL12-dependent phenotypes were significantly reversed by *HOXB5* knockdown (Figure 5E-I). Additionally, we observed similar effects in the *in vivo* liver metastatic assays (Figure 5J-N). These results suggested that HOXB5 was required for CXCL12-mediated CRC metastasis to distant organs.

Our results raised the possibility that there was a CXCL12-HOXB5-ITGB3 signal cascade under CXCL12-mediated CRC invasion and metastasis. To address this issue, we treated Caco-2 cells with CXCR4 inhibitor AMD3100 or ERK inhibitor SCH772984, and conducted *ETS1*/*HOXB5* shRNA knockdown in these cells before CXCL12 treatment. Indeed, depletion of *ETS1*/*HOXB5* mRNA or pharmacological inhibition of CXCR4/ERK significantly impaired CXCL12-induced ITGB3 expression, suggesting the CXCL12-CXCR4-ERK1/2-ETS1-HOXB5 signaling cascade is required for this process (Supplementary Figure S4). To explore the epistasis between ITGB3 and the CXCL12-CXCR4-HOXB5 signaling cascade, we enforced ITGB3 expression in CXCL12-overexpressing Caco-2 cells with simultaneous *HOXB5* shRNA knockdown (Caco-2-CXCL12+LV-shHOXB5) (Supplementary Figure S5A). Results of transwell assays demonstrated that enforced ITGB3 expression partially rescued the decreased migration and invasion of Caco-2-CXCL12 cells mediated by *HOXB5* knockdown (Supplementary Figure S5B). Results of *in vivo* orthotopic CRC assays also showed that ITGB3 overexpression not only reversed the impaired metastatic capacity of Caco-2-CXCL12 cells mediated by *HOXB5* knockdown but also shortened the overall survival time of the nude mice (Supplementary Figure S5C-I). These data collectively demonstrated that ITGB3 was epistatic to the CXCL12-CXCR4-HOXB5 signaling cascade in CRC metastasis.

### AMD3100, a specific CXCR4 antagonist, suppresses HOXB5-mediated CRC invasion and metastasis

**Figure 6:**
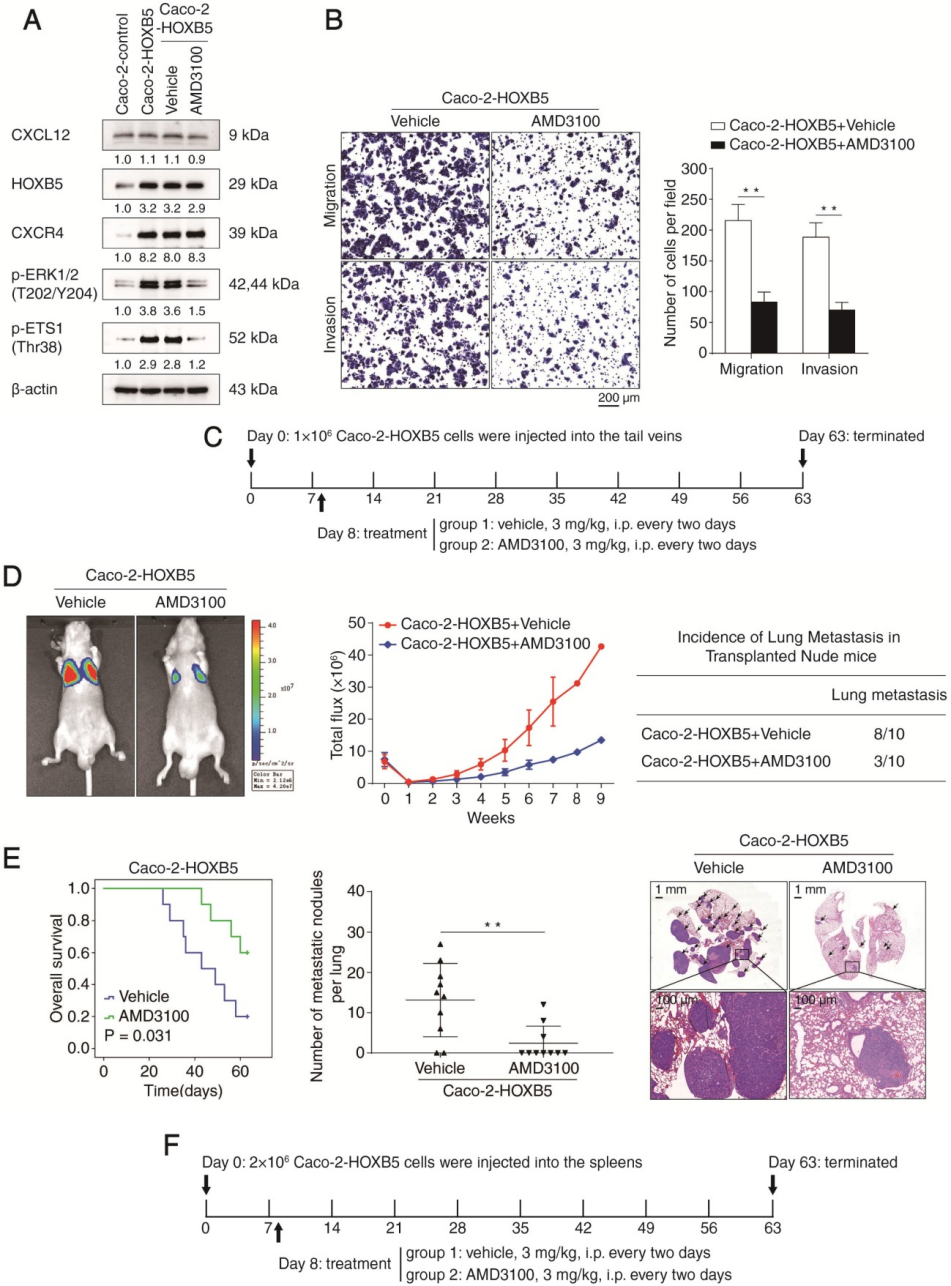

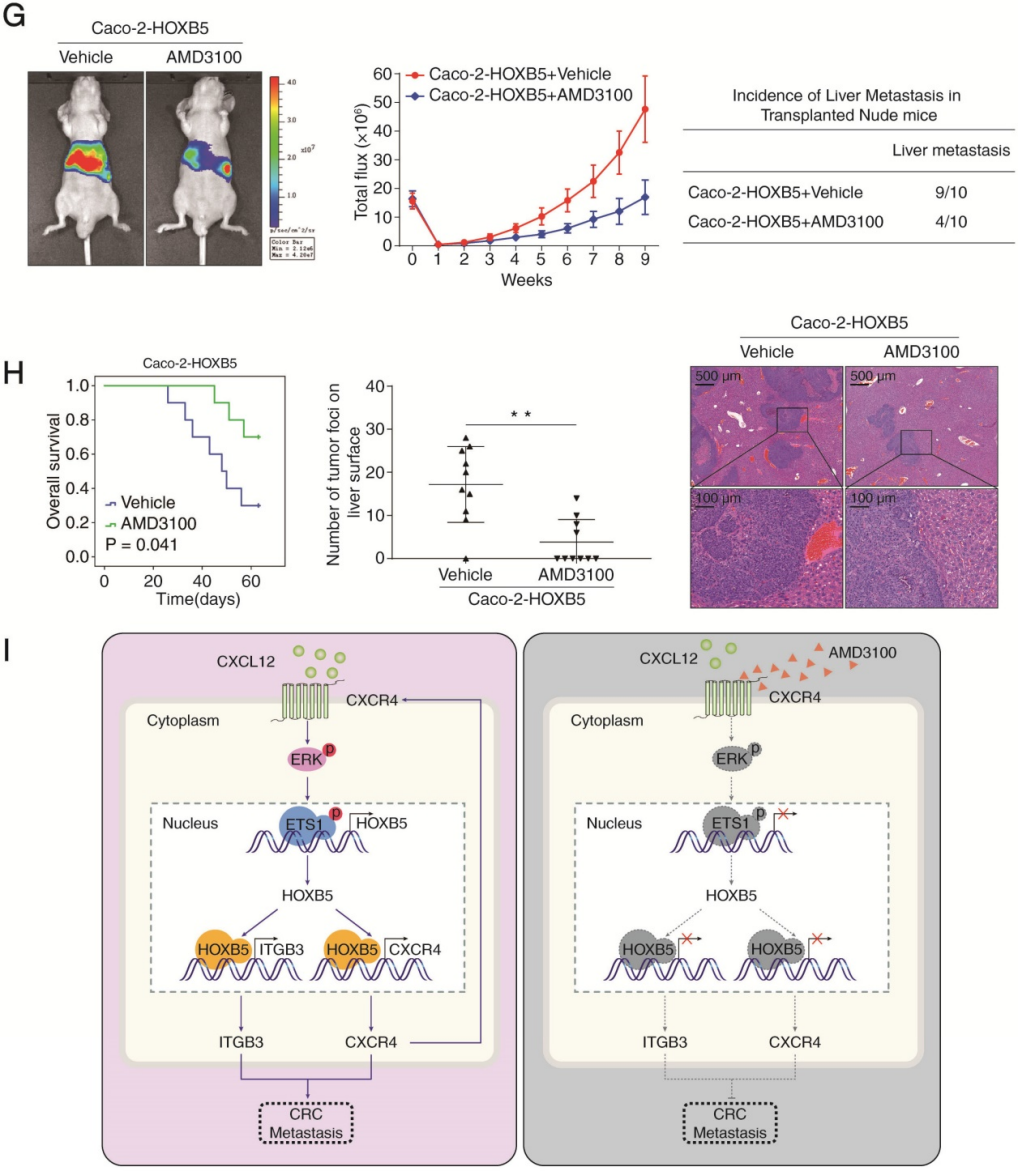
** CXCR4 inhibitor AMD3100 suppresses HOXB5-mediated CRC metastasis. (A)** Caco-2 cells were incubated with vehicle or AMD3100 (1 µg/ml, 24 hours) after lentivirus transfection (LV-HOXB5), then Western blotting assays were conducted to measure the protein levels of CXCL12, HOXB5, CXCR4, p-ERK and p-ETS1 in the indicated cell lines. **(B)** Transwell assays indicated that AMD3100 treatment (1 µg/ml, 24 hours) significantly decreased the migration and invasion of Caco-2-HOXB5 cells. **(C-E)*** In vivo* lung metastatic assays showed that AMD3100 administration significantly inhibited HOXB5-mediated CRC metastasis. Tumor cells were injected into the tail veins of nude mice (n = 10 mice per group). (C) AMD3100 or vehicle (3 mg/kg) was administered intraperitoneally to the nude mice every two days from day 8 after implantation to day 63. (D) Representative bioluminescent imaging at 9 weeks after implantation, the intensity of bioluminescence signals for 9 consecutive weeks and the incidence of lung metastasis were shown (E) Overall survival time of nude mice, the number of lung metastatic nodules and representative images of H&E staining of lung tissues from the two groups. The scale bars represent 1 mm (low magnification) and 100 µm (high magnification). **(F-H)** *In vivo* liver metastatic assays demonstrated that AMD3100 treatment dramatically reduced HOXB5-mediated CRC metastasis. Tumor cells were injected into the spleens of nude mice (n = 10 mice per group). (F) Nude mice were intraperitoneally administered with AMD3100 or vehicle (3 mg/kg) every two days from day 8 after implantation to day 63. (G) Representative bioluminescent imaging at 9 weeks, the bioluminescence signals of the two groups recorded for 9 consecutive weeks and the incidence of liver metastasis were shown. (H) Overall survival time of the nude mice, the number of metastatic nodules in the liver and representative images of H&E staining of liver tissues from the two groups are presented. The scale bars represent 500 µm (low magnification) and 100 µm (high magnification). All the data are shown as the mean ± s.d. *P < 0.05, **P < 0.01. **(I)** A schematic diagram of the role of the CXCL12-HOXB5-CXCR4 positive feedback loop in CRC metastasis. The CXCL12-CXCR4 axis upregulates HOXB5 expression through activating the ERK1/2-ETS1 signaling pathway. HOXB5 overexpression promotes CRC invasion and metastasis by transactivating CXCR4 and ITGB3 expression. The administration of AMD3100, a specific CXCR4 inhibitor, effectively disrupts the CXCL12-HOXB5-CXCR4 feedback loop and inhibits HOXB5-mediated CRC invasion and metastasis.

Given the important roles of the CXCR4/CXCL12 axis in HOXB5-mediated CRC metastasis, we set out to explore whether pharmacological inhibition of CXCR4 could reverse this process. The specific CXCR4 antagonist AMD3100 has been approved by the FDA for the autologous transplantation of bone marrow cells in patients with non-Hodgkin's lymphoma or multiple myeloma 28. The efficacy of AMD3100 treatment was confirmed by the significant reduction of p-ERK1/2 and p-ETS1, a readout for CXCR4 signaling activation (Figure 6A). Next, we investigated whether AMD3100 treatment affected the migration and invasion of Caco-2-HOXB5 cells and the parental Caco-2 cells. Results of transwell assays indicated that AMD3100 treatment at a dose of 1 μg/ml inhibited the migration and invasion of Caco-2-HOXB5 cells but had no effect on Caco-2 cells (Figure 6B, Supplementary Figure S6A). Interestingly, CXCR4 blockade with AMD3100 also reduced endogenous HOXB5 protein levels in several CRC cell lines with relatively high basal levels of HOXB5 (Supplementary Figure S7).

To test whether AMD3100 could inhibit HOXB5-mediated CRC metastasis *in vivo*, xenograft nude mice were intraperitoneally administrated with AMD3100 or vehicle every two days at a dose of 3 mg/kg from day 8 after implantation. In both *in vivo* lung metastatic assays and *in vivo* liver metastatic assays, AMD3100 treatment significantly reduced the intensity of BLI signals, the incidence of metastasis and the number of metastatic nodules while prolonged the overall survival time of the nude mice implanted with Caco-2-HOXB5 cells (Figure 6D-E, 6G-H). Next, we conducted orthotopic CRC assays to examine the effect of AMD3100 treatment on the *in vivo* metastatic capacity of Caco-2-HOXB5 cells and the parental Caco-2 cells. AMD3100 treatment significantly reduced the metastasis burden and improved the prognosis of mice bearing Caco-2-HOXB5 cells but had no apparent effect on the metastasis burden or the prognosis of mice bearing Caco-2 cells (Supplementary Figure S6B-H). These results suggested that targeting CXCR4 signaling with AMD3100 is a promising approach to block HOXB5-mediated CRC invasion and metastasis.

## Discussion

Chemokine CXCL12 and its receptor CXCR4 are promising therapeutic targets for cancer management. A better understanding of CXCR4 regulation and signal transduction is essential for the development of effective CXCR4 targeted therapy. Previous studies demonstrated that transcription factor HOXB5 is upregulated in multiple human cancers and facilitates cancer progression by promoting tumor proliferation and metastasis 5-8, 10. In the present study, we found that the expression level of HOXB5 was significantly elevated in human CRC which positively correlated with poor prognosis. Additionally, HOXB5 overexpression was evident in CRC patients with metastasis compared with CRC patients who did not have metastasis. Furthermore, in metastatic CRC patients, we observed a markedly increased HOXB5 expression in metastatic CRC tissues than primary CRC tissues. These clinical observations strongly suggested that HOXB5 may be a functional driver of CRC progression and metastasis. Interestingly, this hypothesis was further supported by a series of *in vitro* and* in vivo* studies, which demonstrated that overexpression of HOXB5 facilitates CRC invasion and metastasis. In line with this, our data also showed that HOXB5 promoted CRC proliferation and tumor growth. As cancer cell proliferation at primary and metastatic sites are two critical steps in the metastatic cascade, HOXB5-enhanced cell proliferation may be an underlying mechanism for HOXB5-mediated CRC metastasis.

The CXCL12/CXCR4 axis is a key regulator of cancer metastasis and tumor microenvironment 11, 29. At the primary tumor sites, chemokine CXCL12 initiates the early step of metastasis through activating its receptor CXCR4 in a paracrine or autocrine manner 13. When tumor cells enter the circulation, CXCR4-positive tumor cells tend to migrate to common metastasis sites with high level of CXCL12 expression, such as liver, lung, lymph nodes and bones 30. After these cells colonize a distant organ site, the CXCL12/CXCR4 axis provides further support to their outgrowth 11. In human CRC, the CXCL12/CXCR4 signaling pathway accelerates metastasis initiation through multiple mechanisms 17, 27. For example, it promotes the dissemination of CXCR4-positive CRC cells to organs with abundant CXCL12 31, 32, and supports the outgrowth of micro-metastases after distant colonization 33.

The β3 integrin (ITGB3) is known to play an important role in cancer metastasis 34. It regulates multiple biological processes of tumor progression, including reprogramming tumor metabolism, maintaining tumor stemness, promoting angiogenesis, enhancing drug resistance, re-educating tumor microenvironment and facilitating EMT 35. Preclinical studies have found that overexpression of ITGB3 facilitates cancer invasion and metastasis in HCC 36 and ALK-rearranged NSCLC 37, whereas ITGB3 silencing significantly inhibits EMT and metastasis of triple-negative breast cancer 38. Interestingly, the upregulation of ITGB3 expression by reactive oxygen species (ROS) markedly promoted the migration and invasion capacities of CRC cells 21, and its antagonist significantly decreased metastasis burden and improved survival in CRC xenograft models 39. These studies indicate that ITGB3 is another key regulator of CRC invasion and metastasis.

In this study, we demonstrated that both CXCR4 and ITGB3 were transcriptional targets of the homeobox transcription factor HOXB5. Knockdown of *CXCR4* and *ITGB3* significantly impaired CRC invasion and metastasis modulated by HOXB5 overexpression, whereas the decreased invasive and metastatic abilities of CRC cells mediated by *HOXB5* knockdown was readily rescued by overexpression of CXCR4 or ITGB3. Our work showed that HOXB5 expression positively correlated with ITGB3 and CXCR4 expression in human CRC tissues. Importantly, the subgroups of patients with HOXB5/ITGB3 or HOXB5/CXCR4 co-expression had the highest recurrence rates and shortest overall survival times in our cohorts. These results suggested that overexpression of HOXB5 may promote CRC invasion and metastasis through the transactivation of CXCR4 and ITGB3.

In addition to functional characterization of CXCR4/ITGB3 in HOXB5-mediated CRC metastasis, we also established the existence of a positive feedback loop between HOXB5 and the CXCR4 signaling. In the tumor microenvironment, signal transduction modulated by chemokines and their cognate receptors can affect tumor cell proliferation, stemness and angiogenesis, which ultimately alter the prognosis of patients 40. It has been shown that chemokines-chemokine receptor signaling pathways mediate the tumor-stroma interaction in advanced and metastatic CRC and promote cancer invasion and metastasis 41. Consistently, CXCL12 was known to exert pro-growth and pro-metastasis functions in CRC through the CXCR4 signaling pathways 42. In line with this, our work demonstrated that CXCL12 can upregulate HOXB5 expression in human CRC through the CXCR4-ERK1/2-ETS1 signaling cascade. Reciprocally, HOXB5-mediated CXCR4 induction increased the sensitivity of CRC cells to CXCL12 stimulation and formed a positive feedback loop in promoting CRC invasion and metastasis. Therefore, targeting this positive feedback loop may provide a promising strategy to reduce CRC metastasis.

Finally, we set out to take a pharmacological approach to break this CXCL12-HOXB5-CXCR4 positive feedback. Recently, several antagonists targeting the CXCL12/CXCR4 axis were under clinical development 12. Among them, the CXCR4 inhibitor AMD3100 came to our attention due to its efficacy and safety profiles. As a mobilizer of hematopoietic stem cells (HSCs), AMD3100 has been approved by the US FDA for autologous transplantation in patients with non-Hodgkin's lymphoma or multiple myeloma 28. Recently, AMD3100 has been rigorously investigated in many clinical trials based on its capacity to suppress the CXCR4 signaling in preclinical models of multiple human cancers including lung cancer 43, osteosarcoma 44, prostate cancer 45, gastric cancer 46, hepatocellular carcinoma 47, breast cancer 48 and ovarian cancer 49. Importantly, we found that AMD3100 markedly inhibited HOXB5-induced CRC invasion and metastasis via suppressing the CXCL12-CXCR4-ERK1/2-ETS1 signaling cascade. Our results indicated that targeting CXCR4 signaling with AMD3100 may be a promising therapeutic approach to reduce CRC metastasis and improve patient survival.

In conclusion, we demonstrated that upregulation of HOXB5 facilitated CRC metastasis through transactivating CXCR4 and ITGB3. Through the CXCR4-ERK1/2-ETS1 signaling cascade, CXCL12 increased HOXB5 expression which formed a CXC12-HOXB5-CXCR4 positive feedback loop to promote CRC metastasis. The administration of CXCR4 inhibitor AMD3100 effectively disrupted this positive feedback loop and suppressed HOXB5-mediated CRC metastasis. Our study raises the possibility that metastatic CRC patients may receive clinical benefit from CXCR4 inhibition. This possibility should be assessed with clinical trials of CXCR4 inhibition in combination with chemotherapy or immunotherapy.

## Supplementary Material

Supplementary figures and tables.

## Figures and Tables

**Table 1:** Correlation between HOXB5 expression and clinicopathological characteristics of CRCs in two independent cohorts of human CRC tissues

Clinicopathological variables	Cohort I (n = 334)		*p* value	Cohort II (n = 390)		*p* value
	Tumor HOXB5 expression			Tumor HOXB5 expression		
	Negative(n = 187)	Positive (n = 147)		Negative (n = 209)	Positive (n = 181)	
**Age**	66.43 (10.26)	65.67 (12.43)	0.541	67.96 (11.32)	66.94 (11.82)	0.389
**Sex**						
Female	83	69		88	89	
Male	104	78	0.659	121	92	0.185
**Tumor location**						
Right colon	90	71		87	78	
Left colon	53	64		90	78	
Rectum	44	12	< 0.001	32	25	0.914
**Tumor size**						
< 5 cm	86	58		80	69	
≥ 5 cm	101	89	0.266	129	112	1
**Tumor differentiation**						
Well or moderate	151	75		149	75	
Poor	36	72	< 0.001	60	106	< 0.001
**Tumor invasion**						
T1	5	2		12	4	
T2	21	9		14	10	
T3	124	93		147	126	
T4	37	43	0.097	36	41	0.205
**Lymph node metastasis**						
Absent	138	47		175	51	
Present	49	100	< 0.001	34	130	< 0.001
**Distant metastasis**						
Absent	169	100		199	119	
Present	18	47	< 0.001	10	62	< 0.001
**AJCC stage**						
Stage I	25	10		16	4	
Stage II	111	33		156	43	
Stage III	33	57		27	74	
Stage IV	18	47	< 0.001	10	60	< 0.001

**Table 2:** Univariate and multivariate analysis of factors associated with survival and recurrence in two independent cohorts of human CRC tissues

Clinical Variables	Time To Recurrence		Overall Survival	
	HR (95% CI)	*P* value	HR (95% CI)	*P* value
***Cohort I (n = 334)***				
**Univariate analysis**				
Age	0.993 (0.980-1.006)	0.280	0.993 (0.980-1.006)	0.271
Sex (female versus male)	1.232 (0.924-1.642)	0.156	1.160 (0.866-1.552)	0.319
Tumor size (< 5 versus ≥ 5 cm)	0.797 (0.594-1.069)	0.130	0.791 (0.587-1.065)	0.123
Tumor differentiation (well/moderate versus poor)	0.141 (0.104-0.192)	< 0.001	0.147 (0.108-0.200)	< 0.001
Tumor invasion (T1-T3 versus T4)	0.346 (0.255-0.467)	< 0.001	0.355 (0.261-0.482)	< 0.001
Lymph node metastasis (absent versus present)	0.072 (0.049-0.105)	< 0.001	0.075 (0.051-0.110)	< 0.001
Distant metastasis (absent versus present)	0.110 (0.078-0.155)	< 0.001	0.113 (0.080-0.159)	< 0.001
AJCC stage(I-II versus III-IV)	0.068 (0.046-0.101)	< 0.001	0.071 (0.048-0.105)	< 0.001
HOXB5 expression (negative versus positive)	0.321 (0.238-0.432)	< 0.001	0.311 (0.230-0.421)	< 0.001
**Multivariate analysis**				
Tumor differentiation (well/moderate versus poor)	0.761 (0.518-1.118)	0.164	0.807 (0.547-1.191)	0.281
Tumor invasion (T1-T3 versus T4)	0.657 (0.470-0.918)	0.014	0.669 (0.474-0.945)	0.022
Lymph node metastasis (absent versus present)	0.394 (0.151-1.029)	0.057	0.392 (0.148-1.036)	0.059
Distant metastasis (absent versus present)	0.459 (0.308-0.684)	< 0.001	0.447 (0.299-0.669)	< 0.001
AJCC stage (I-II versus III-IV)	0.268 (0.097-0.741)	0.011	0.279 (0.099-0.784)	0.015
HOXB5 expression (negative versus positive)	0.661 (0.482-0.907)	0.010	0.612 (0.444-0.845)	0.003
***Cohort II (n = 390)***				
**Univariate analysis**				
Age	0.998 (0.988-1.009)	0.774	1.000 (0.989-1.011)	0.951
Sex (female versus male)	1.070 (0.847-1.351)	0.569	1.116 (0.880-1.415)	0.367
Tumor size (< 5 versus ≥ 5 cm)	0.901 (0.709-1.146)	0.396	0.877 (0.686-1.122)	0.297
Tumor differentiation (well/moderate versus poor)	0.469 (0.370-0.593)	< 0.001	0.449 (0.353-0.571)	< 0.001
Tumor invasion (T1-T3 versus T4)	0.605 (0.461-0.796)	< 0.001	0.607 (0.459-0.803)	< 0.001
Lymph node metastasis (absent versus present)	0.193 (0.150-0.248)	< 0.001	0.172 (0.132-0.222)	< 0.001
Distant metastasis (absent versus present)	0.130 (0.095-0.178)	< 0.001	0.111 (0.081-0.154)	< 0.001
AJCC stage(I-II versus III-IV)	0.179 (0.138-0.230)	< 0.001	0.159 (0.122-0.207)	< 0.001
HOXB5 expression (negative versus positive)	0.374 (0.294-0.474)	< 0.001	0.355 (0.278-0.453)	< 0.001
**Multivariate analysis**				
Tumor differentiation (well/moderate versus poor)	0.808 (0.621-1.053)	0.114	0.815 (0.622-1.069)	0.140
Tumor invasion (T1-T3 versus T4)	0.693 (0.522-0.920)	0.011	0.705 (0.528-0.943)	0.018
Lymph node metastasis (absent versus present)	1.442 (0.671-3.100)	0.348	1.222 (0.568-2.627)	0.608
Distant metastasis (absent versus present)	0.370 (0.262-0.525)	< 0.001	0.326 (0.229-0.465)	< 0.001
AJCC stage (I-II versus III-IV)	0.186 (0.083-0.413)	< 0.001	0.195 (0.087-0.436)	< 0.001
HOXB5 expression (negative versus positive)	0.697 (0.531-0.914)	0.009	0.689 (0.522-0.909)	0.008

## Abbreviations

CRC: colorectal cancer
HOXB5: Homeobox B5
CXCR4: C-X-C motif chemokine receptor 4
ITGB3: integrin subunit beta 3
CXCL12: C-X-C motif chemokine ligand 12
IHC: immunohistochemistry
mRNA: messenger RNA
shRNA: short hairpin RNA
BLI: bioluminescent imaging
ChIP: chromatin immunoprecipitation
H&E: hematoxylin and eosin
ERK: extracellular regulated protein kinase
PI3K: phosphoinositide 3-kinase
mTOR: mechanistic target of rapamycin kinase
PKC: protein kinase C
PKA: protein kinase A
SP1: Sp1 transcription factor
ETS1: ETS Proto-Oncogene 1, Transcription Factor
ELK1: ETS Transcription Factor ELK1
siRNA: small interfering RNA
EMT: epithelial-mesenchymal transition
