# Supplementary material for: CXCL12-mediated HOXB5 overexpression facilitates Colorectal Cancer metastasis through transactivating CXCR4 and ITGB3
Source: Theranostics. 2021 Jan 1;11(6):2612–33. doi: 10.7150/thno.52199 (PMC7806482; doi:10.7150/thno.52199)
Supplement: Supplementary file 1 — Supplementary figures and tables. [file thnov11p2612s1.pdf]

Supplementary material

Methods

Cell culture

All cells used in this study were obtained from the American Tissue Type Culture Collection. Each cell line was tested and authenticated by their manufacturers. The cells were cultured in Dulbecco's modified Eagle's medium (Gibco, Thermo Fisher Scientific, Cambridge, MA, USA) supplemented with 10% fetal bovine serum (FBS, Gibco), 100 μg/ml penicillin, and 100 μg/ml streptomycin (Gibco) in a 5% CO_2 atmosphere at 37 ℃. These above cell lines were authenticated by short tandem repeats (STRs) DNA profiling. All cells were tested for mycoplasma contamination before use with the Universal Mycoplasma Detection Kit (ATCC 30-1012K) and were not contaminated by mycoplasma.

Plasmid construction

All plasmid generation was carried out based on previously published standard procedures [1]. Briefly, for example, the HOXB5 gene complete CDS construct, pCMV-HOXB5, was generated by using cDNA from human PBMCs. It was generated with forward and reverse primers incorporating EcoRI and XhoI sites at the 5' and 3'-ends, respectively. The final polymerase chain reaction (PCR) product was cloned into the EcoRI and XhoI sites of the pCMV-Tag2B vector. Besides, human genomic DNA was used to produce the CXCR4 promoter construct (-4700/+212) CXCR4, which corresponds to the -4700 to +212 sequence (relative to the

transcriptional start site) of the 5'-flanking region of the human CXCR4 gene. Forward and reverse primers containing the SacI and XhoI sites at the 5' and 3'-ends, respectively, were used to build the construct. The final PCR product was cloned into the SacI and XhoI sites of the pGL3-Basic vector (Promega). Similarly, constructs containing a deletion of the 5'-flanking region of the CXCR4 promoter, [(-4558/+212) CXCR4, (-3076/+212) CXCR4, (-1117/+212) CXCR4 and (-428/+212) CXCR4], were built based on the (-4700/+212) CXCR4 construct as the template. The QuikChange II Site-Directed Mutagenesis Kit (Stratagene, CA, USA) was used to mutate the HOXB5 binding sites of the CXCR4 promoter. The sequence integrity of all constructs was verified by gene sequencing. All promoter constructs used in this experiment were generated similarly. All primers are listed in Supplementary Table S4.

Construction of lentivirus and stable cell lines

Lentivirus production was performed according to a previously described protocol [1]. Lentiviral vectors encoding shRNAs were generated using PLKO.1-TRC (Addgene) and designated as LV-shHOXB5, LV-shCXCR4, LV-shITGB3, and LV-shcontrol. "LV-shcontrol" is a non-target shRNA control. The vector "pLKO.1-puro Non-Target shRNA Control Plasmid DNA" (purchased from Sigma, SHC016) contains an shRNA insert that does not target any known genes from any species. Three short hairpin RNAs (shRNAs) sequences were presented in Supplementary Table S5. Lentiviral vectors encoding the human HOXB5, CXCR4,

ITGB3 and CXCL12 genes were constructed in PLKO.1-TRC (Addgene) and designated as LV-HOXB5, LV-CXCR4, LV-ITGB3 and LV-CXCL12. An empty vector was used as the negative control and was designated as LV-control. The lentivirus and cell infection were produced according to the pLKO.1 lentiviral vector protocol recommended by Addgene. Briefly, the lentiviral plasmid and packaging plasmids pMD2. G and psPAX2 (Addgene plasmid #12259 and #12260) were transfected into HEK-293T cells with transfection reagent (Lipofectamine®3000, Thermo Fisher Scientific) and OPTI-MEM media (Invitrogen, Waltham, MA, USA). The lentiviruses were harvested twice on days 4 and 5. Viruses were filtered with a 0.45-μm filter and stored at -80 ℃. Lentiviral infection of target cells was performed in cell culture media with 5 μg/ml polybrene (Sigma H9268). Seventy-two hours after infection, cells were selected for 2 weeks using 2.5 μg/ml puromycin (OriGene). Selected pools of cells were used for the following experiments.

Transient transfection

A total of 1×10^5 serum-starved cells were plated in each well of a 24-well plate and allowed to attach for 12-24 hours. Then, a mixture of Lipofectamine 3000 (Invitrogen, USA) containing 0.02 μg of the pRL-TK plasmids, 0.18 μg of the promoter reporter plasmids and 0.6 μg of the expression vector plasmids was used to cotransfect cells for 5 hours based on the manufacturer's instructions. The cells were then washed and incubated with 1% FBS-supplemented fresh medium for 48 hours.

Luciferase reporter assay

The Dual Luciferase Assay (Promega, USA) was used to quantify luciferase activity following the manufacturer's instructions. Transfected cells were subjected to cell lysis in a culture dish with lysis buffer. Subsequent lysates were transferred to an Eppendorf microcentrifuge before being centrifuged for 1 minute at maximum speed. The efficiency of transfection was normalized to Renilla activity, and relative luciferase activity was quantified with a Modulus TM TD20/20 Luminometer (Turner Biosystems, USA).

***In vitro* migration and invasion assays**

The invasive and migratory capabilities of each cell line were assessed with an 8-μm pore, 24-well Transwell plate (Corning, USA). For invasion assays, chamber inserts were first coated with 60 μL of Matrigel (Corning, 200 mg/mL) and left to dry overnight under sterile conditions. The next day, the uppermost chamber was plated at a cell density of 1×10^5. For cell migration assays, the upper chamber, which was lined with a noncoated membrane, was plated with cells at a density of 5×10^4. Each assay was repeated thrice, and three different inserts were used to obtain a mean cell number in five fields per membrane.

Western blot analyses

Proteins from lysed cells were fractionated by SDS-PAGE and transferred to nitrocellulose membranes. Nonspecific binding sites were blocked with 5% milk in

TBST (120 mM Tris–HCl (pH 7.4), 150 mM NaCl, and 0.05% Tween 20) for 1 hour at room temperature. Blots were incubated with a specific antibody overnight at 4 ℃. Western blotting of β-actin on the same membrane was used as a loading control. The membranes were incubated with primary antibodies overnight at 4 ℃. The membranes were then washed with PBS 3 times and incubated with an HRP-conjugated secondary antibody. Proteins were visualized using a Immobilon™ Western Chemiluminescent HRP substrate(Millipore, USA).

The primary antibodies used are listed below.

Antibodies	Source	Concentration
anti-HOXB5	Abcam, ab109375	1/1000
anti-CXCR4	Abcam, ab124824	1/100
anti-ITGB3	Cell signaling technology, 13166S	1/1000
anti-CXCL12	Cell signaling technology, 3530S	1/1000
anti-p-ERK1/2(T202/Y204)	Cell Signaling Technology, 4370S	1/2000
anti-ERK1/2	Cell Signaling Technology, 9102S	1/1000
anti-p-Akt(Ser-473)	Cell Signaling Technology, 4060S	1/2000
anti-Akt(pan)	Cell Signaling Technology, 4685S	1/1000
anti-p-p70S6K(T389)	Cell Signaling Technology, 9205S	1/1000
anti-p70S6K	Cell Signaling Technology, 9202S	1/1000
anti-p-PKCα(T638)	Abcam, ab32502	1/5000
anti-PKCα	Cell Signaling Technology, 2056S	1/1000
anti-p-PKA C(Thr197)	Cell Signaling Technology, 5661S	1/1000

anti-p-PKA C-α	Cell Signaling Technology, 5842S	1/1000
anti-p-ETS1(T38)	Invitrogen, 44-1104G	1/1000
anti-ETS1	Cell Signaling Technology, 14069S	1/1000
anti-MMP2	Abcam, ab92536	1/3000
anti-MMP7	Abcam, ab207299	1/1000
anti-MMP13	Abcam, ab39012	1/3000
anti-β-actin	Cell Signaling Technology, 4967S	1/2000

Real-time qPCR

The RNeasy Plus Mini Kit (50) kit (Qiagen, Hilden, Germany) was used to extract total RNA, which was then reverse transcribed with the Advantage RT-for-PCR Kit (Qiagen) in accordance with the manufacturer's protocols. The target sequence was amplified with real-time PCR with the SYBR Green PCR Kit (Qiagen). The cycling parameters used were 95 ℃ for 15 s, 55-60 ℃ for 15 s, and 72 ℃ for 15 s for 45 cycles. Melting curve analyses were performed, and Ct values were determined during the exponential amplification phase of real-time PCR. SDS 1.9.1 software (Applied Biosystems, Massachusetts, USA) was used to evaluate amplification plots. The $2^{-\Delta\Delta Ct}$ method was used to determine relative fold changes in target gene expression in cell lines, which was normalized to expression levels in corresponding control cells (defined as 1.0). The equation used was $2^{-\Delta\Delta Ct}$ ($\Delta Ct = \Delta Ct^{target} - \Delta Ct^{GAPDH}$; $\Delta\Delta Ct = \Delta Ct^{expressing\ vector} - \Delta Ct^{control\ vector}$). When calculating relative expression levels in surgically extracted CRC samples, relative fold changes in target

gene expression were normalized to expression values in normal colon epithelial tissues (defined as 1.0) using the following equation: $2^{-\Delta\Delta Ct}$ ($\Delta\Delta Ct = \Delta Ct^{tumor} - \Delta Ct^{nontumor}$). All experiments were performed in duplicate. Supplementary Table S4 lists all sequences of all primers used.

CCK-8 assay

For the CCK-8 assay, cells were seeded into 96-well plates at a density of 1000 cells in 100 μl of complete medium per well. At each time point, the original medium was replaced with CCK-8 solution (TransDetect Cell Counting Kit, Transgene, Beijing, China) and complete medium mixed at a 1:9 ratio, and the cells were then incubated at 37 °C for 2 h. The absorbance of each sample was recorded at 450 nm using a microplate reader (Tecan Group, Ltd, Zürich, Switzerland) and each sample was measured three times.

Colony formation assay

Transfected cells (1000 per well) were cultured in 6-well plates. After 14 days of culture, the cells formed stable colonies. The cell colonies were fixed with 4% paraformaldehyde for 10 minutes and then stained with a crystal violet solution for 10 minutes. Colonies containing more than 50 cells were counted and each group included three replicates.

***In vivo* tumor growth in the xenograft model**

Six-week-old BALB/C nude mice were cared for and maintained based on our institution’s protocols for ethical animal care. The Committee on the Use of Live Animals in Teaching and Research (CULATR) of the Fourth Military Medical University approved all animal experiments. For the in *vivo* growth assay, suspended treated cells were subcutaneously injected into the flank of each mouse (6 mice per group, 1×10^6 cells in 150 μl of PBS per mouse). The mice were weighed and the tumor size was measured using vernier calipers. The tumor volume was calculated using the following equation: $V (mm^3) = 0.5 \times L (mm) \times W^2 (mm^2)$. After 4 weeks, all mice were sacrificed. Then, tumor weight measured.

Quantification and Statistical Analysis

The quantitative data were compared between groups using the Student's t-test. Categorical data were analyzed using the Fisher’s exact test. The cumulative recurrence and survival rates were determined using the Kaplan-Meier method and log-rank test. The Cox proportional hazards model was used to determine the independent factors that influence survival and recurrence based on the variables that had been selected from the univariate analyses. A value of $p < 0.05$ was considered to be significant. All the analyses were performed using the SPSS software (version 19.0).

References

1. Huang W, Chen Z, Shang X, Tian D, Wang D, Wu K, et al. Sox12, a direct target of FoxQ1, promotes hepatocellular carcinoma metastasis through up-regulating Twist1 and FGFBP1. Hepatology. 2015; 61: 1920-33.

Supplementary Figure S1

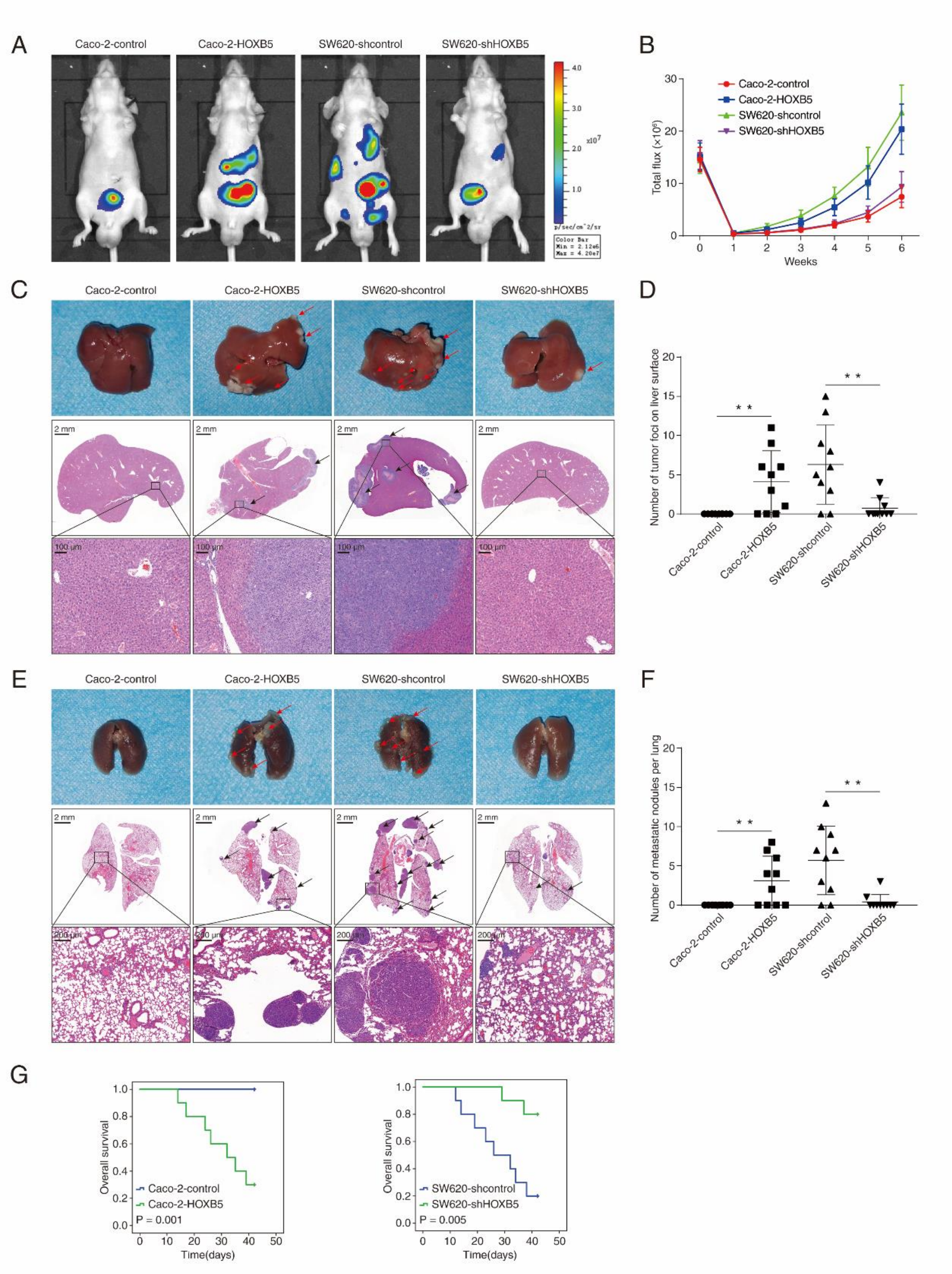

Supplementary Figure S1. *In vivo* orthotopic CRC models indicate HOXB5 promotes CRC metastasis.

(A) Representative bioluminescent images of the different groups at 6 weeks after

orthotopic implantation were shown.

(B) The intensity of bioluminescence signals of the different groups was recorded for 6 consecutive weeks.

(C) Representative pictures of gross liver and representative images of H&E staining of liver tissues from the different groups were shown. The scale bars represent 2 mm (low magnification) and 100 μm (high magnification).

(D) The number of liver metastatic nodules in the different groups was calculated.

(E) Representative pictures of gross lung and representative images of H&E staining of lung tissues in each group. The scale bars represent 2 mm (low magnification) and 200 μm (high magnification).

(F) The number of lung metastatic foci in each group was counted.

(G) The overall survival time of the nude mice in the different groups.

All the data are shown as the mean ± s.d. * $P < 0.05$ ** $P < 0.01$

Supplementary Figure S2

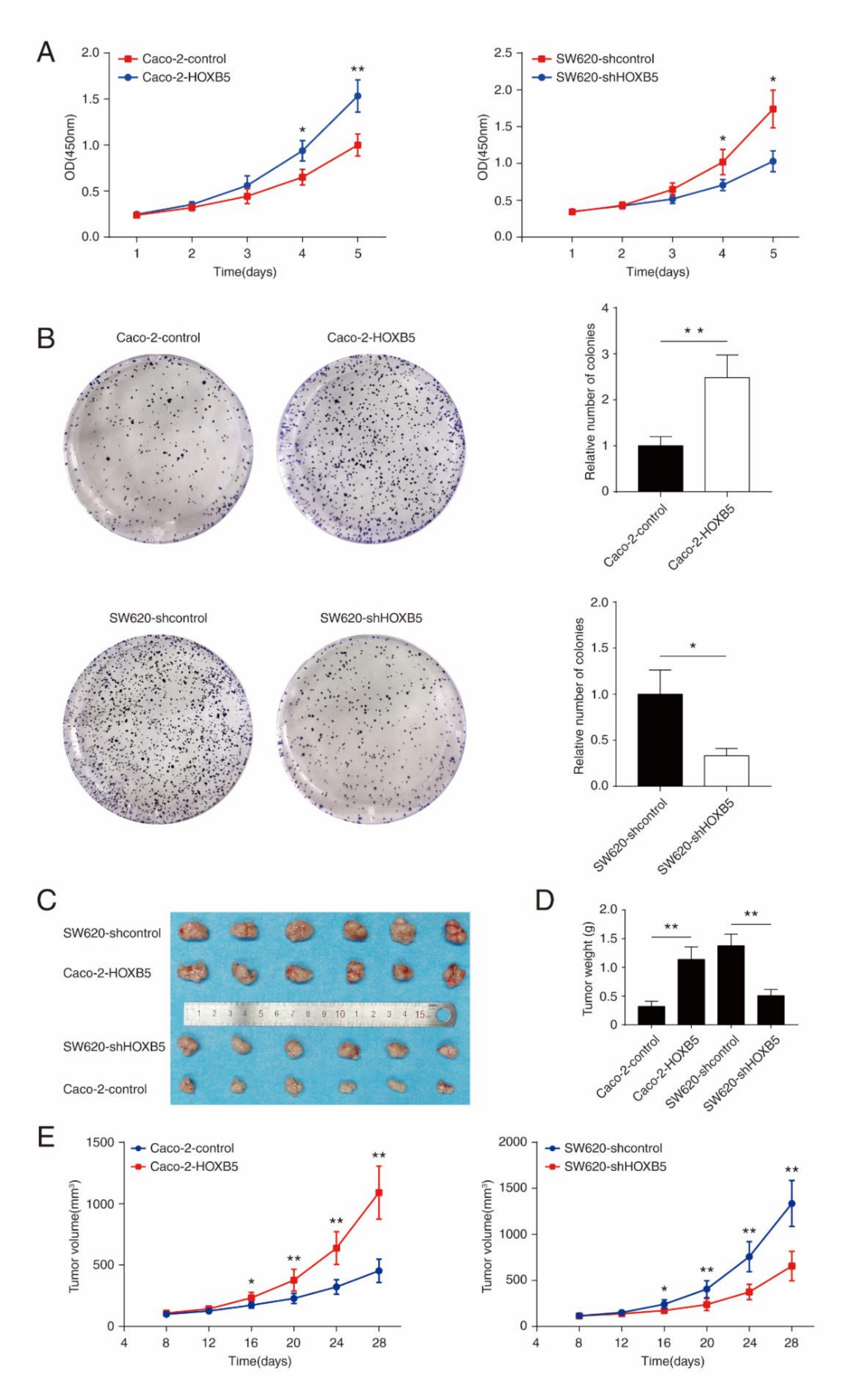

Supplementary Figure S2. HOXB5 promotes CRC cell proliferation and tumor

growth.

(A) The proliferation capacities of the indicated CRC cell lines were measured by a Cell Counting Kit-8 (CCK8) assay. The data are shown as the mean ± s.d. of three experiments. * $P < 0.05$ ** $P < 0.01$

(B) The indicated CRC cells were subjected to colony formation assays. One thousand cells were seeded into 6-well plates with the medium. Two weeks later, the number of colonies > 40 um in each group was counted. Statistical comparison of the number of colonies in the indicated groups was performed. The data are shown as the mean ± s.d. of three experiments. * $P < 0.05$ ** $P < 0.01$

(C-E) Subcutaneous xenograft tumor models were established to measure the tumorigenicity of the indicated CRC cell lines. Each experimental group contained 6 nude mice. (C) The gross picture of subcutaneous tumors of the different groups. (D) The weight of tumors in the indicated groups was measured and compared. (E) The growth curves of tumors in the indicated groups were shown. The data are shown as the mean ± s.d. * $P < 0.05$ ** $P < 0.01$

Supplementary Figure S3

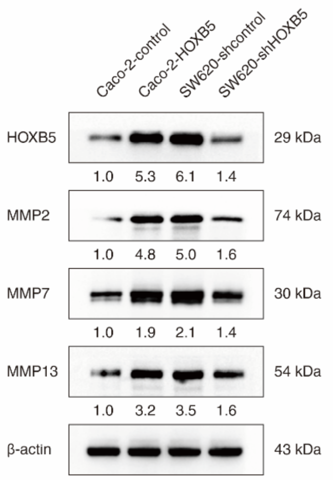

Supplementary Figure S3. Western blotting assays were conducted to quantify the protein levels of HOXB5, MMP2, MMP7, and MMP13 in the indicated CRC cell lines.

Supplementary Figure S4

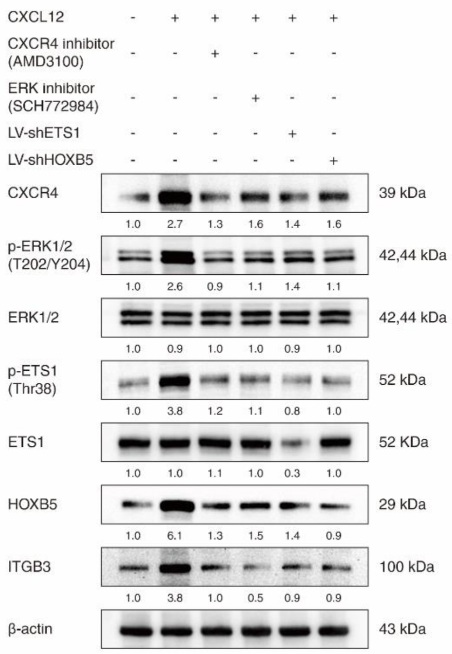

Supplementary Figure S4. The CXCL12-CXCR4-ERK1/2-ETS1-HOXB5 signaling pathway upregulates ITGB3 expression in CRC cells.

Caco-2 cells were cultured with CXCR4 inhibitor AMD3100 or ERK inhibitor SCH772984, or infected with lentivirus LV-shETS1 or LV-shHOXB5 individually before CXCL12 treatment (100 ng/ml, 24 hours). Western blotting assays were then performed to detect the expression levels of relevant proteins in the CXCR4-ERK1/2-ETS1-HOXB5-ITGB3 signaling pathway.

Supplementary Figure S5

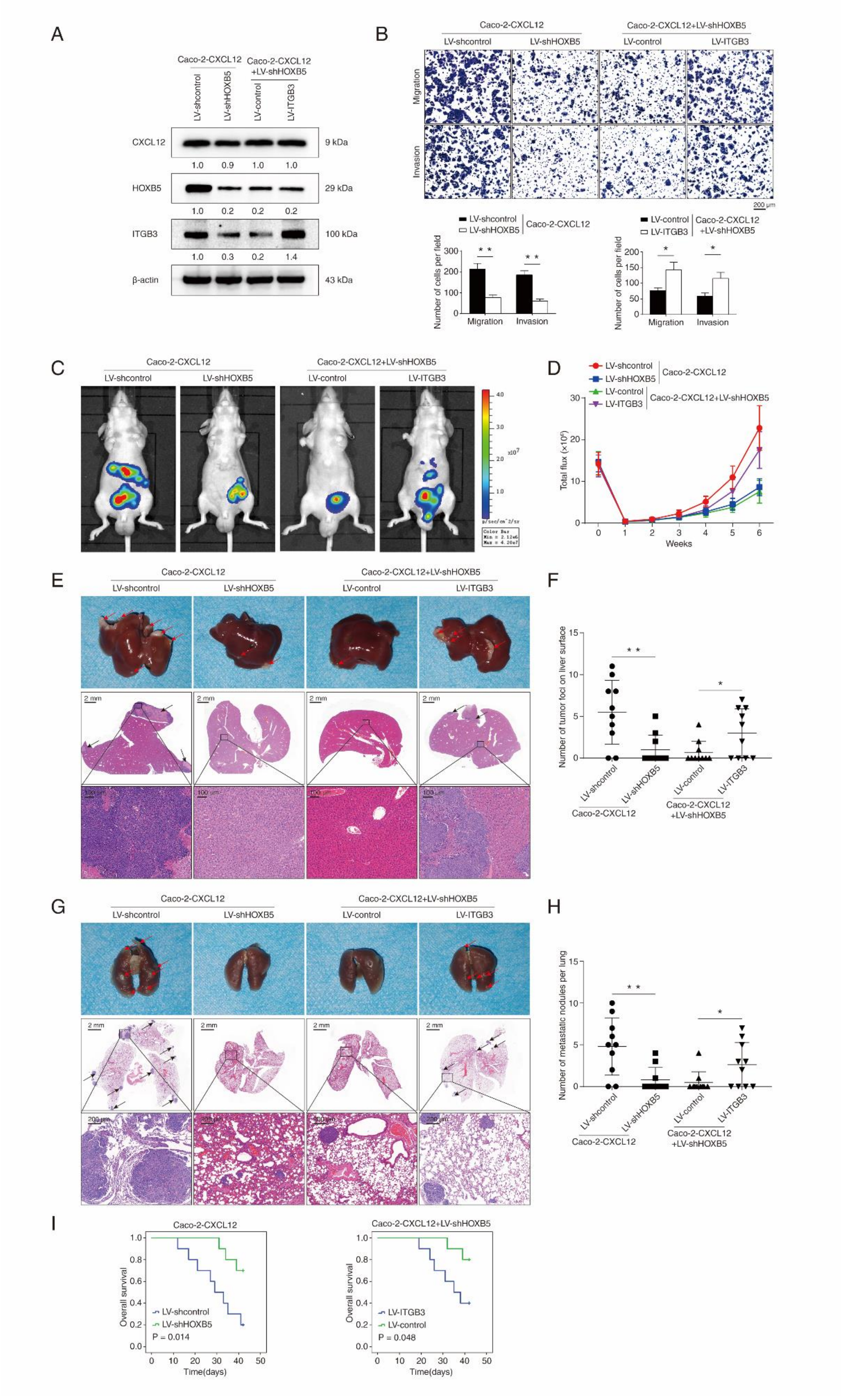

Supplementary Figure S5. The involvement of ITGB3 in HOXB5-mediated CRC invasion and metastasis is controlled by the CXCL12-CXCR4 axis.

(A) ITGB3 expression was upregulated in CXCL12-overexpressing Caco-2 cells with HOXB5 knockdown (Caco-2-CXCL12 + LV-shHOXB5) by lentiviral transfection. Western blotting analysis of the protein levels of CXCL12, HOXB5 and ITGB3 in the indicated CRC cell lines.

(B) Transwell assays showed that ITGB3 overexpression partially rescued the decreased migration and invasion of Caco-2-CXCL12 cells induced by HOXB5 knockdown.

(C-I) *In vivo* orthotopic CRC models indicated that ITGB3 overexpression effectively reversed the impaired metastatic capacity caused by HOXB5 knockdown in Caco-2-CXCL12 cells. (C) Representative bioluminescent images of the different groups at 6 weeks after orthotopic implantation. (D) The intensity of bioluminescence signals for 6 consecutive weeks of the different groups. (E) Representative pictures of gross liver and representative images of H&E staining of liver tissues in each group. The scale bars represent 2 mm (low magnification) and 100 μm (high magnification). (F) The number of liver metastatic nodules in the different groups. (G) Representative pictures of gross lung and representative images of H&E staining of lung tissues from the different groups. The scale bars represent 2 mm (low magnification) and 200 μm (high magnification). (H) The number of lung metastatic foci in each group. (I) The overall survival time of the nude mice in the different groups.

Supplementary Figure S6

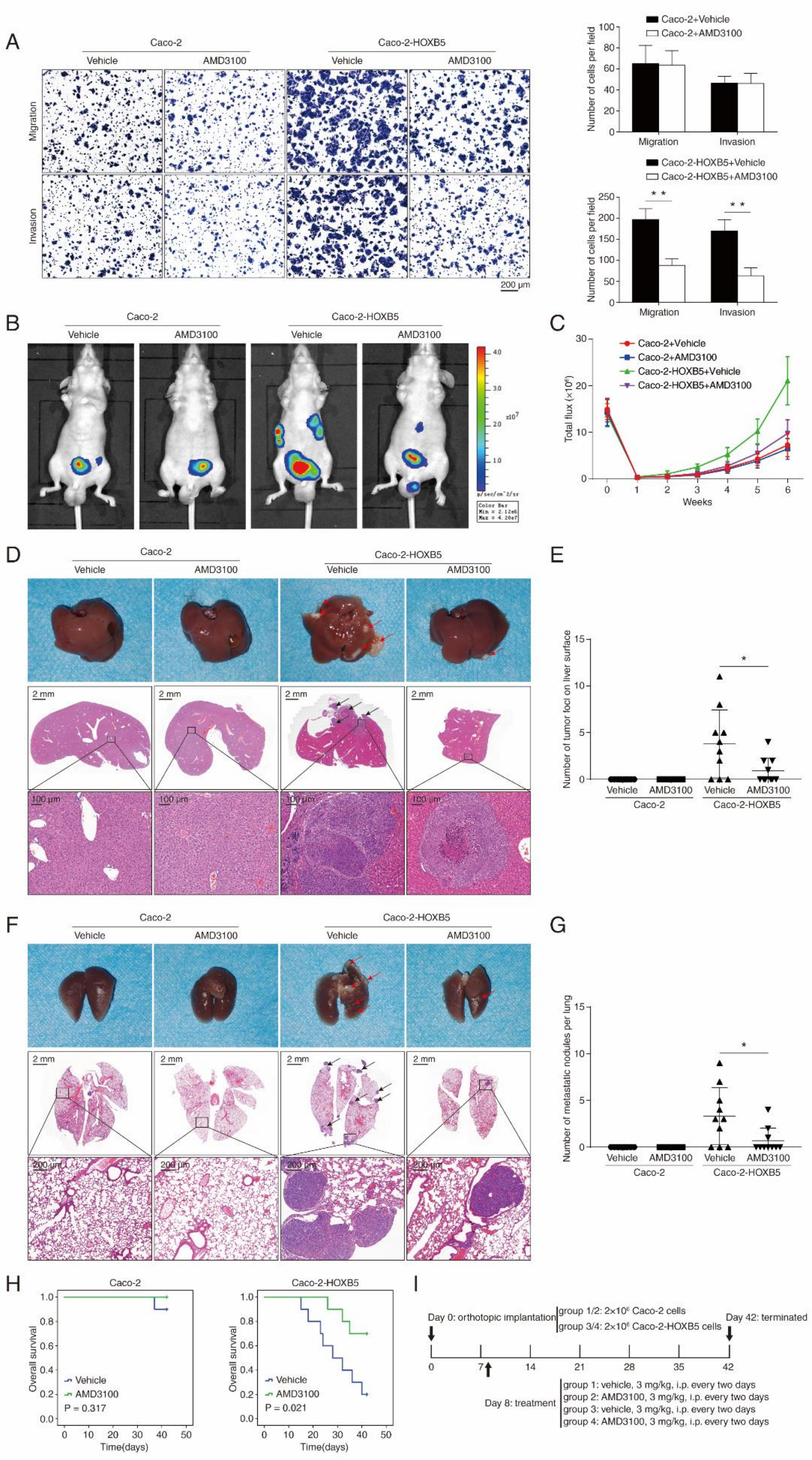

Supplementary Figure S6. AMD3100 administration significantly suppresses HOXB5-mediated CRC invasion and metastasis.

(A) Transwell assays were performed under AMD3100 or vehicle incubation at a dose of 1 μg/ml for 24 hours. AMD3100 treatment showed no effect on the migration and invasion of Caco-2 cells but significantly reduced the migration and invasion of Caco-2-HOXB5 cells.

(B-H) *In vivo* orthotopic CRC models indicate AMD3100 administration suppresses HOXB5-mediated CRC metastasis. (B) Representative bioluminescent images of the different groups at 6 weeks after orthotopic implantation. (C) The intensity of bioluminescence signals for 6 consecutive weeks of the different groups. (D) Representative pictures of gross liver and representative images of H&E staining of liver tissues in each group. The scale bars represent 2 mm (low magnification) and 100 μm (high magnification). (E) The number of liver metastatic nodules in the different groups. (F) Representative pictures of gross lung and representative images of H&E staining of lung tissues from the different groups. The scale bars represent 2 mm (low magnification) and 200 μm (high magnification). (G) The number of lung metastatic foci in each group. (H) The overall survival time of the nude mice in the different groups.

(I) Schematic overview of experimental processing was shown. AMD3100 or vehicle (3 mg/kg) was administered intraperitoneally to the nude mice every two days from day 8 after implantation to day 42.

All the data are shown as the mean ± s.d. * $P < 0.05$ ** $P < 0.01$

Supplementary Figure S7

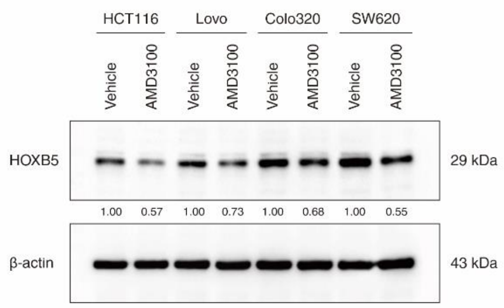

Supplementary Figure S7. Western blotting analysis of HOXB5 expression in the indicated CRC cells after the cells were treated with AMD3100.

Supplementary Figure S8

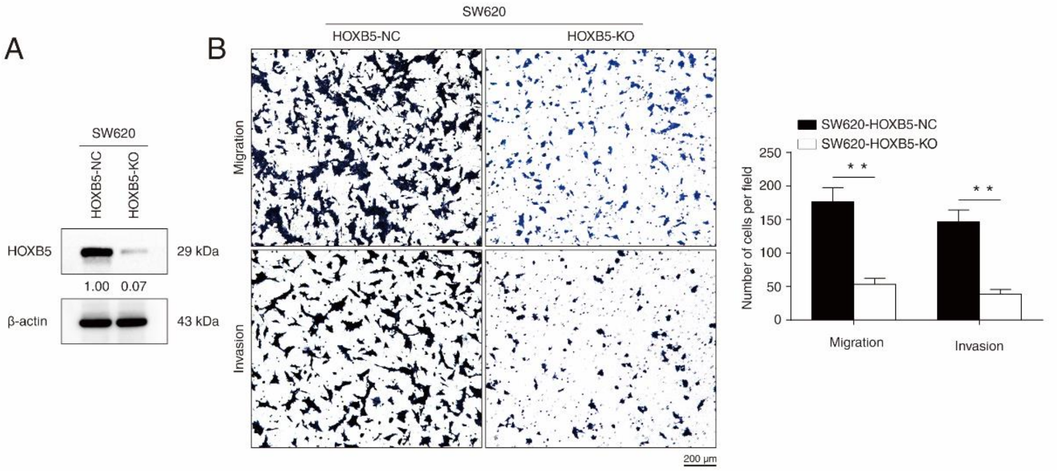

Supplementary Figure S8. Depletion of HOXB5 using CRISPR Knockout technology inhibits the migration and invasion of SW620 cells.

(A) SW620 cells were infected with lentivirus LV-NC and LV-HOXB5 KO, and were selected with puromycin for 7 days. Then the pool cells were used for Western blotting analysis.

(B) Transwell assays were used to detect the migration and invasion abilities of the indicated cells.

Supplementary Table S1. List of genes differentially expressed in Caco-2-HOXB5 versus Caco-2-control cells using a human metastasis PCR array

**Symbol**	**Caco-2-HOXB5 vs. Caco-2-control**	**Gene function**	**Description**
CXCR4	6.92	signal transduction, promotes invasion	Chemokine (C-X-C motif) receptor 4
ITGB3	6.23	participates in cell adhesion	Integrin, beta 3 (platelet glycoprotein IIIa, antigen CD61)
MTA1	5.34	promotes metastasis	Metastasis associated 1
ETV4	5.15	transcription factor, promotes proliferation	Ets variant 4
MYCL	4.89	transcription factor, promotes proliferation	V-myc myelocytomatosis viral oncogene homolog 1, lung carcinoma derived (avian)
MMP2	4.51	decomposes, protein hydrolysate and promotes metastasis	Matrix metallopeptidase 2
MGAT5	4.11	promotes metastasis	Mannosyl (alpha-1,6-)-glycoprotein beta-1,6-N-acetyl-glucosaminyltransferase
MMP13	3.93	decomposes, protein hydrolysate and promotes metastasis	Matrix metallopeptidase 13 (collagenase 3)
CHD4	3.68	chromatin assemble and modification	Chromodomain helicase DNA binding protein 4
VEGFA	3.51	promotes proliferation, metastasis; inhibits apoptosis	Vascular endothelial growth factor A
SRC	3.08	promotes proliferation	V-src sarcoma (Schmidt-Ruppin A-2) viral oncogene homolog (avian)
TGFB1	2.94	inhibits or promotes proliferation, promotes metastasis	Transforming growth factor, beta 1
COL4A2	2.76	the component of extracellular matrix	Collagen, type IV, alpha 2
EPHB2	2.33	signal transduction, promotes invasion	EPH receptor B2
MMP7	2.25	decomposes, protein hydrolysate and promotes metastasis	Matrix metallopeptidase 7
FAT1	2.10	participates in cell adhesion	FAT tumor suppressor homolog 1 (Drosophila)
FN1(fibronectin)	1.98		Fibronectin 1
CD44	1.95	cell adhesion and stroma attachment	CD44 molecule (Indian blood group)
HRAS	1.90	promotes proliferation	V-Ha-ras Harvey rat sarcoma viral oncogene homolog
TCF20	1.87	transcription factor	Transcription factor 20 (AR1)
MCAM	1.82	participates in cell adhesion	Melanoma cell adhesion molecule
DENR	1.82	promotes proliferation	Density-regulated protein
CXCR2	1.77	signal transduction, promotes invasion	Chemokine (C-X-C motif) receptor 2
MMP11	1.75	decomposes, protein hydrolysate and promotes metastasis	Matrix metallopeptidase 11 (stromelysin 3)

SYK	1.71	promotes proliferation	Spleen tyrosine kinase
IGF1	1.69	promotes proliferation	Insulin-like growth factor 1 (somatomedin C)
FGFR4	1.62	promotes invasion	Fibroblast growth factor receptor 4
MET	1.58	Proto-oncogene, promotes cell proliferation	Met proto-oncogene (hepatocyte growth factor receptor)
MMP10	1.55	protein hydrolysate, promotes metastasis	Matrix metallopeptidase 10 (stromelysin 2)
SERPINE1(PAI1)	1.51		Serpin peptidase inhibitor, clade E (nexin, plasminogen activator inhibitor type 1), member 1
CST7	1.51	inhibits cysteine proteinase	Cystatin F (leukocystatin)
MMP9	1.46	decomposes, protein hydrolysate and promotes metastasis	Matrix metallopeptidase 9
CTSK	1.43	protein hydrolysate	Cathepsin K
ITGA7	1.39	participates in cell adhesion	Integrin, alpha 7
CTNNA1	1.38	participates in cell adhesion	Catenin (cadherin-associated protein), alpha 1, 102kDa
FLT4	1.31	transcription factor, promotes proliferation	Fms-related tyrosine kinase 4
RORB	1.27	participates in regulate of transcription	RAR-related orphan receptor B
CCL7	1.22	promotes invasion and migration	Chemokine (C-C motif) ligand 7
EWSR1	1.21	transcription factor, promotes oncogenesis	Ewing sarcoma breakpoint region 1
SMAD4	1.19	cell signal transduction	SMAD family member 4
CXCL12	1.17	participates in cell adhesion	Chemokine (C-X-C motif) ligand 12
HTATIP2	1.14	positively regulates transcription	HIV-1 Tat interactive protein 2, 30kDa
RPSA	1.12	participates in cell adhesion	Ribosomal protein SA
CTSL	1.12	protein hydrolysate	Cathepsin L1
TSHR	1.10	promotes proliferation	Thyroid stimulating hormone receptor
MYC	1.09	promotes proliferation	V-myc myelocytomatosis viral oncogene homolog (avian)
CDH6	1.06	osteosis, cell adhesion	Cadherin 6, type 2, K-cadherin (fetal kidney)
METAP2	1.05	protein hydrolysate and modification	Methionyl aminopeptidase 2
SMAD2	1.05	cell signal transduction	SMAD family member 2
MMP3	1.03	decomposes, protein hydrolysate and promotes metastasis	Matrix metallopeptidase 3 (stromelysin 1, progelatinase)
CDH11	1.02	involves in the metastatic process	Cadherin 11, type 2, OB-cadherin (osteoblast)
HGF	1.02	participates in proteolysis, promotes proliferation	Hepatocyte growth factor (hepapoietin A; scatter factor)
NR4A3	1.01	transcription factor, promotes proliferation	Nuclear receptor subfamily 4, group A, member 3
TRPM1	1.01	calcium channels	Transient receptor potential cation channel, subfamily M, member 1
NME1	1	negatively regulates proliferation and participates in cell adhesion	Non-metastatic cells 1, protein (NM23A) expressed in

BRMS1	-1.05	inhibits metastasis and tumor growth	Breast cancer metastasis suppressor 1
KRAS	-1.08	cell signal transduction, proliferation	V-Ki-ras2 Kirsten rat sarcoma viral oncogene homolog
IL1B	-1.09	inhibits or promotes proliferation	Interleukin 1, beta
RB1	-1.13	negative regulation of cell reproduction	Retinoblastoma 1
TP53	-1.15	induces apoptosis and cell differentiation, inhibits proliferation	Tumor protein p53
HPSE	-1.18	hydrolyses protein	Heparanase
IL18	-1.21	promotes cell proliferation	Interleukin 18 (interferon-gamma-inducing factor)
MDM2	-1.24	negative regulation of cell proliferation	Mdm2 p53 binding protein homolog (mouse)
PLAUR	-1.25	activator of plasminogen	Plasminogen activator, urokinase receptor
SSTR2	-1.28	inhibits proliferation	Somatostatin receptor 2
NME4	-1.28	inhibits proliferation	Non-metastatic cells 4, protein expressed in
APC	-1.30		Adenomatous polyposis coli
KISS1	-1.34	suppresses metastasis	KiSS-1 metastasis-suppressor
SET	-1.37	inhibits histone acetylation	SET nuclear oncogene
CDH1(E-cadherin)	-1.46	inhibits tumor metastasis	Cadherin 1, type 1, E-cadherin (epithelial)
GNRH1	-1.52	inhibits cell proliferation	Gonadotropin-releasing hormone 1 (luteinizing-releasing hormone)
CDKN2A(p16)	-1.64	negative regulation of cell cycle	Cyclin-dependent kinase inhibitor 2A (melanoma, p16, inhibits CDK4)
NF2	-1.68	inhibits proliferation	Neurofibromin 2 (merlin)
PNN	-1.75	inhibits proliferation	Pinin, desmosome associated protein
CD82	-1.96	metastasis suppressor	CD82 molecule
TIMP2	-2.12	inhibits metastasis	TIMP metallopeptidase inhibitor 2
TIMP4	-2.25	inhibits metastasis	TIMP metallopeptidase inhibitor 4
CTBP1	-2.31	inhibits cell proliferation	C-terminal binding protein 1
MTSS1	-2.52	inhibits metastasis and proliferation	Metastasis suppressor 1
TNFSF10	-2.78	induces apoptosis, inhibits proliferation	Tumor necrosis factor (ligand) superfamily, member 10
FXYD5	-2.83	negative regulation of cell adhesion	FXYD domain containing ion transport regulator 5
PTEN	-3.17	inhibits proliferation and metastasis	Phosphatase and tensin homolog
KISS1R	-3.35	suppresses metastasis	KISS1 receptor
TIMP3	-3.68	induces apoptosis, inhibits metastasis	TIMP metallopeptidase inhibitor 3

Supplementary Table S2. Correlation between CXCR4 expression and clinicopathological characteristics of CRCs in two independent cohorts of human CRC tissues

Clinicopathological variables		Cohort I (n = 334) Tumor CXCR4 expression Negative (n = 164)	Cohort I (n = 334) Tumor CXCR4 expression Positive (n = 170)	p Value	Cohort II (n = 390) Tumor CXCR4 expression Negative (n = 200)	Cohort II (n = 390) Tumor CXCR4 expression Positive (n = 190)	p Value
Age		66.69(10.90)	65.53(11.59)	0.347	66.95(11.13)	68.06(11.98)	0.342
Sex	female	65	87		91	86	
	male	99	83	0.037	109	104	1
Tumor location	right colon	79	82		82	83	
	left colon	45	72		90	78	
	rectum	40	16	< 0.001	28	29	0.732
Tumor size	＜ 5cm	74	70		79	70	
	⩾ 5cm	90	100	0.508	121	120	0.604
Tumor differentiation	well or moderate	134	92		130	94	
	poor	30	78	< 0.001	70	96	0.002
Tumor invasion	T1	6	1		10	6	
	T2	19	11		16	8	
	T3	111	106		137	136	
	T4	28	52	0.004	37	40	0.316
Lymph node metastasis	absent	124	61		149	77	
	present	40	109	< 0.001	51	113	< 0.001
Distant metastasis	absent	150	119		183	135	
	present	14	51	< 0.001	17	55	< 0.001
AJCC stage	Stage I	25	10		14	6	
	Stage II	98	46		134	65	
	Stage III	27	63		37	64	
	Stage IV	14	51	< 0.001	15	55	< 0.001

Supplementary Table S3. Correlation between ITGB3 expression and clinicopathological characteristics of CRCs in two independent cohorts of human CRC tissues

		Cohort I (n = 334)			Cohort II (n = 390)		
		Tumor ITGB3 expression			Tumor ITGB3 expression		
Clinicopathological variables		Negative (n = 174)	Positive (n = 160)	p Value	Negative (n = 213)	Positive (n = 177)	p Value
Age		66.32(11.25)	65.86(11.29)	0.706	67.46(11.09)	67.51(12.12)	0.967
Sex	female	79	73		95	82	
	male	95	87	1	118	95	0.76
Tumor location	right colon	93	68		84	81	
	left colon	51	66		97	71	
	rectum	30	26	0.064	32	25	0.443
Tumor size	＜ 5cm	71	73		88	61	
	⩾ 5cm	103	87	0.379	125	116	0.175
Tumor differentiation	well or moderate	143	83		140	84	
	poor	31	77	< 0.001	73	93	<0.001
Tumor invasion	T1	7	0		13	3	
	T2	22	8		13	11	
	T3	110	107		153	120	
	T4	35	45	0.002	34	43	0.042
Lymph node metastasis	absent	127	58		160	66	
	present	47	102	< 0.001	53	111	< 0.001
Distant metastasis	absent	159	110		195	123	
	present	15	50	< 0.001	18	54	< 0.001
AJCC stage	Stage I	27	8		14	6	
	Stage II	98	46		144	55	
	Stage III	34	56		38	63	
	Stage IV	15	50	< 0.001	17	53	< 0.001

Supplementary Table S4. Primer sequences used in the study

Primer name	Primer sequences	Enzyme
Primers for real-time PCR:		
HOXB5 sense:	5'-AGCTCCAGCGCCAATTTCA-3'	
HOXB5 antisense:	5'-TCCGGCCCGGTCATATCAT-3'	
CXCR4 sense:	5’-ACTACACCGAGGAAATGGGCT-3’	
CXCR4 antisense:	5’-CCCACAATGCCAGTTAAGAAGA-3’	
ITGB3 sense:	5’-AGTAACCTGCGGATTGGCTTC-3’	
ITGB3 antisense:	5’-GTCACCTGGTCAGTTAGCGT-3’	
GAPDH sense:	5’-CTGGGCTACACTGAGCACC-3’	
GAPDH antisense:	5’-AAGTGGTCGTTGAGGGCAATG-3’	
Primers for pCMV-HOXB5 construct:		
Sense	5’-TGTTGAATTCATGAGCTCGTACTTTGTAAAC-3’	EcoRI
Antisense	5’-TTTTCTCGAGTCAGGGCTGGAAGGCGCTGC-3’	XhoI
Primers for CXCR4 promoter construct:		
(-4700/+212)CXCR4 sense:	5’-TATAGAGCTCAGGAAAACAATGACAAGCCTA-3’	SacI
(-4558/+212)CXCR4 sense:	5’-TATAGAGCTCCATAAAGCCACACAAGCACCT-3’	SacI
(-3076/+212)CXCR4 sense:	5’-TATAGAGCTCAGGTCAGTTGCACATAACAAGC-3’	SacI
(-1117/+212)CXCR4 sense:	5’-TATAGAGCTCAGATCGCTTTAAACGTCTGACC-3’	SacI
(-428/+212)CXCR4 sense:	5’-TATAGAGCTCTACTTCCAGCATTGCCGCCTA-3’	
Antisense:	5’-ATATCTCGAGTACGGGTACCTCCAATGTCC-3’	XhoI
Primers for CXCR4 promoter site-directed mutagenesis:		
HOXB5 binding site:		
binding site 4 mutation sense:	5’-TTCACTCACTCcggcATTTCCTAAAG-3’	
binding site 4 mutation antisense:	5’-CTTTAGGAAATgccgGAGTGAGTGAA-3’	
binding site 3 mutation sense:	5’-ATTGCACTGTTcggcAAGTGGCTGTT-3’	
binding site 3 mutation antisense:	5’-AACAGCCACTTgccgAACAGTGCAAT-3’	
binding site 2 mutation sense:	5’-ATTGTTCTTTTcggcATCTGCTTCCT-3’	
binding site 2 mutation antisense:	5’-AGGAAGCAGATgccgAAAAGAACAAT-3’	
binding site 1 mutation sense:	5’-AAGTATCTCCTcggcATCTGCACCTG-3’	
binding site 1 mutation antisense:	5’-CAGGTGCAGATgccgAGGAGATACTT-3’	
Primers used for ChIP in the CXCR4 promoter:		
distant region sense:	5’-ATGGCTATCATCAACACCTT-3’	
distant region antisense:	5’-TGTAATCCGCCCATCTCG-3’	
binding site 4/3 sense:	5’-TAAAGCCACACAAGCACCT-3’	
binding site 4/3 antisense:	5’-AGCCATCCTGGTAAGTCA-3’	
binding site 2 sense:	5’-AGCTGGTCTTTTCAAATCCG-3’	
binding site 2 antisense:	5’-GCACGATGTCTAAAACCTG-3’	
binding site 1 sense:	5’-ACTGGTCGCTAAATTACTCT-3’	
binding site 1 antisense:	5’-CAAGCTTCCAACGCCAT-3’	
Primers for ITGB3 promoter construct:		
(-4465/+107)ITGB3 sense:	5’-TATAGCTAGCAGTTCAAGGCTACAATCAGCTA-3’	NheI
(-4054/+107)ITGB3 sense:	5’-TATAGCTAGCCACTGTGAATCAAATGCCAC-3’	NheI

(-3050/+107)ITGB3 sense:	5’-TATAGCTAGCATAGGCATTAGCTACCACACC-3’	NheI
(-2078/+107)ITGB3 sense:	5’-TATAGCTAGCAACCTGCTTCACATCATGGTC-3’	NheI
(-1675/+107)ITGB3 sense:	5’-TATAGCTAGCTGGACTAGCTCCTTTGCCTT-3’	NheI
(-522/+107)ITGB3 sense:	5’-TATAGCTAGCCGGAAAACCAAACTAAGGCAAG-3’	NheI
Antisense:	5’-ATATAGATCTTCACTCACCTCCTACGCCAAC-3’	BglII

Primers for ITGB3 promoter site-directed mutagenesis:

HOXB5 binding site:

binding site 5 mutation sense:	5’-TGTTGAATGTTcggcAATAACTGTAT-3’
binding site 5 mutation antisense:	5’-ATACAGTTATTgccgAACATTCAACA-3’
binding site 4 mutation sense:	5’-GCCACAGTAATcggcGTTGCAGTTAA-3’
binding site 4 mutation antisense:	5’-TTAACTGCAACgccgATTACTGTGGC-3’
binding site 3 mutation sense:	5’-CTGTGTTTGATcggcATGATGTAGAA-3’
binding site 3 mutation antisense:	5’-TTCTACATCATgccgATCAAACACAG-3’
binding site 2 mutation sense:	5’-AACACAACCATCcggcACTATACTAAA-3’
binding site 2 mutation antisense:	5’-TTTAGTATAGTgccgGATGGTTGTGTT-3’
binding site 1 mutation sense:	5’-CTCACTTGCACcggcATGTCATGTTT-3’
binding site 1 mutation antisense:	5’-AAACATGACATgccgGTGCAAGTGAG-3’

Primers used for ChIP in the ITGB3 promoter:

distant region sense:	5’-TTGCACCACTGTACTCC-3’
distant region antisense:	5’-GACACTATGCCTAGCC-3’
binding site 5/4 sense:	5’-GTGAATCAAATGCCACA-3’
binding site 5/4 antisense:	5’-TGCCAGGTTTATTCACT-3’
binding site 3 sense:	5’-ATAGGCATTAGCTACCACAC-3’
binding site 3 antisense:	5’-CCAGCTTCCCCTAACGTTC-3’
binding site 2/1 sense:	5’-ACACACATGCAAACGAGGT-3’
binding site 2/1 antisense:	5’-CTAGCCCAAGCCCTAGCAA-3’

Primers for HOXB5 promoter construct:

(-1739/+465)HOXB5 sense:	5’-TATAGCTAGCTTCTCTAGCCTTTCCTCGT-3’	NheI
(-1002/+465)HOXB5 sense:	5’-TATAGCTAGCTCCCGCTTTACAAACTCT-3’	NheI
(-284/+465)HOXB5 sense:	5’-TATAGCTAGCGCCAAAGCCAACCTTC-3’	NheI
basic HOXB5	5’-TATAGCTAGCACAGGGTTATAACGACCAC-3’	NheI
antisense:	5’-ATATAGATCTCCTCGTCTATTTCGGTGA-3’	BglII

Primers for HOXB5 promoter site-directed mutagenesis:

ELK1/ETS1 binding site mutation sense:	5’-CAAGGAGGTCCatcgGGGGATTTCGA-3’
ELK1/ETS1binding site mutation antisense:	5’-TCGAAATCCCCcgatGGACCTCCTTG-3’
ETS1 binding site mutation sense:	5’-CCTTCCCCCATccaaGCCTCCAATTC-3’
ETS1 binding site mutation antisense:	5’-GAATTGGAGGCttggATGGGGGAAGG-3’
ELK1 binding site mutation sense:	5’-GGCGCTGTGGAttaaTAACAGCAGTT-3’
ELK1 binding site mutation antisense:	5’-AACTGCTGTTAttaaTCCACAGCGCC-3’
SP1 binding site mutation sense:	5’-AGAGGCTAACCttgaCCACAAGAAAG-3’
SP1 binding site mutation antisense:	5’-CTTTCTTGTGGtcaaGGTTAGCCTCT-3’

Primers used for ChIP in the HOXB5 promoter:

distant region sense:	5’-CTCTCTCCTCAACCCC-3’

distant region antisense:	5’-TCGCTCCAGCCTTTATCTCA-3’
binding site 3 sense:	5’-CTTTATTCCTCCGCCCTC-3’
binding site 3 antisense:	5’-TCACTCGCAGCTAGGCA-3’
binding site 2/1 sense:	5’-TCCTGCTCTGTCACTCC-3’
binding site 2/1 antisense:	5’-ACAATTATTGCAACGGGTTT-3’

Supplementary Table S5. Knockdown shRNA sequences used in this study

TRC number	Sequence
HOXB5	
shRNA-1	CCGGGAAGAAGGACAACAAATTGAACTCGAGTTCAATTTGTTGTCCTTCTTCTTTTTG
shRNA-2	CCGGCGGCTACAATTACAATGGGATCTCGAGATCCCATTGTAATTGTAGCCGTTTTT
shRNA-3	CCGGCAGCGCCAATTTCACCGAAATCTCGAGATTTCGGTGAAATTGGCGCTGTTTTT
CXCR4	
TRCN0000004052	CCGGGCTGCCTTACTACATTGGGATCTCGAGATCCCAATGTAGTAAGGCAGCTTTTT
ITGB3	
TRCN0000003238	CCGGCTCATATAGCATTGGACGGAACTCGAGTTCCGTCCAATGCTATATGAGTTTTT
